# The developmental origin and the specification of the adrenal cortex in humans and cynomolgus monkeys

**DOI:** 10.1126/sciadv.abn8485

**Published:** 2022-04-20

**Authors:** Keren Cheng, Yasunari Seita, Taku Moriwaki, Kiwamu Noshiro, Yuka Sakata, Young Sun Hwang, Toshihiko Torigoe, Mitinori Saitou, Hideaki Tsuchiya, Chizuru Iwatani, Masayoshi Hosaka, Toshihiro Ohkouchi, Hidemichi Watari, Takeshi Umazume, Kotaro Sasaki

**Affiliations:** 1 Institute for Regenerative Medicine, University of Pennsylvania, Philadelphia, PA 19104, USA.; 2 Department of Pathology and Laboratory Medicine, University of Pennsylvania, Philadelphia, PA 19104, USA.; 3 Bell Research Center for Reproductive Health and Cancer, Nagoya 460-0003, Japan.; 4 Department of Obstetrics and Gynecology, Hokkaido University Graduate School of Medicine, Sapporo 060-8638, Japan.; 5 Department of Pathology, Sapporo Medical University Graduate School of Medicine, Sapporo 060-8556, Japan.; 6 Department of Anatomy and Cell Biology, Graduate School of Medicine, Kyoto University, Kyoto 606-8501, Japan.; 7 Institute for the Advanced Study of Human Biology (ASHBi), Kyoto University, Kyoto 606-8501, Japan.; 8 Center for iPS Cell Research and Application (CiRA), Kyoto University, Kyoto 606-8507, Japan.; 9 Research Center for Animal Life Science, Shiga University of Medical Science, Otsu 520-2192, Japan.; 10 Fukuzumi Obstetrics and Gynecology Hospital, Sapporo 062-0043, Japan.; 11 Ohkouchi Obstetrics and Gynecology Hospital, Sapporo 060-0062, Japan.

## Abstract

Development of the adrenal cortex, a vital endocrine organ, originates in the adrenogonadal primordium, a common progenitor for both the adrenocortical and gonadal lineages in rodents. In contrast, we find that in humans and cynomolgus monkeys, the adrenocortical lineage originates in a temporally and spatially distinct fashion from the gonadal lineage, arising earlier and more anteriorly within the coelomic epithelium. The adrenal primordium arises from adrenogenic coelomic epithelium via an epithelial-to-mesenchymal transition, which then progresses into the steroidogenic fetal zone via both direct and indirect routes. Notably, we find that adrenocortical and gonadal lineages exhibit distinct HOX codes, suggesting distinct anterior-posterior regionalization. Together, our assessment of the early divergence of these lineages provides a molecular framework for understanding human adrenal and gonadal disorders.

## Teaser

Specification of the adrenal cortex occurs in adrenogenic coelomic epithelium independent of gonadogenesis in humans and monkeys.

## INTRODUCTION

The adrenal cortex is the major source of steroid hormones that drive a plethora of critical physiologic functions. Accordingly, developmental aberrancies in adrenal cortex formation drive various congenital and adult-onset diseases. Traditionally, rodent models have been used to assess the cellular and genetic mechanisms driving mammalian adrenal development. However, critical differences between rodents and humans exist in adrenal structure, steroidogenic function, and adrenal development (*1*, *2*). Mutated genes implicated in human adrenal anomalies do not always result in a similar phenotype in mice, or vice versa (*1*). For example, mutations in *NR5A1*, a master transcription factor of steroidogenesis, severely affect adrenal development in mice but rarely manifest as adrenal defects in humans (*1*, *3*). Thus, the translational applicability of mechanisms driving adrenal development in murine models needs to be interpreted with caution.

Mammalian gonads (ovaries and testes) also produce steroid hormones and commonalities exist between the adrenal cortex and gonads in both their steroid synthetic pathways and developmental origin. Lineage tracing studies in mice reveal that the adrenal cortex and gonads originate from a common progenitor, the adrenogonadal primordium (*4*–*9*). The adrenogonadal primordium expresses GATA4 and WT1, along with NR5A1, and is thought to be established through thickening of coelomic epithelium (CE), which is a derivative of the posterior intermediate mesoderm (*6*–*8*). Thereafter, the mediodorsal portion of the adrenogonadal primordium separates and migrates dorsally to become the adrenal cortex, whereas the ventral portion becomes the gonads (*6*, *7*, *9*). In humans, the gonads and the adrenal cortex are first recognized as morphologically distinct structures at approximately 33 days after conception [Carnegie stage (CS) 15], at which time the adrenal cortex is recognized as a condensed blastematous structure, medial to the mesonephros, referred to as the adrenal primordium (*10*–*13*). However, because of the lack of genetic tracing tools or appropriate markers to differentiate emerging gonadal and adrenocortical lineages, how and when these lineages are specified and segregated remain poorly understood in humans.

In both mice and humans, after formation of the adrenal primordium, the adrenocortical lineage forms morphologically and functionally discrete zones: the inner fetal zone and outer definitive zone (*5*, *14*). In humans and nonhuman primates, androgens produced by the fetal zone promote the formation of the fetoplacental unit, thereby playing a pivotal role in fetal development and pregnancy maintenance (*2*). The fetal zone regresses postnatally, whereas the definitive zone eventually gives rise to the adult adrenal cortex (*2*). Lineage tracing studies in mice have suggested that the fetal zone and definitive zone originate from common progenitors at the early stage of adrenal primordium (*5*). However, the molecular events accompanying the bifurcation of the fetal zone and definitive zone from the earlier progenitor have not been characterized in humans. Through high-resolution lineage trajectory mapping, we now find that adrenocortical specification in primates occurs in a spatially and temporarily distinct manner from gonadogenesis, thus providing essential insight for understanding human adrenogenesis and gonadogenesis.

## RESULTS

### Specification of the gonads in the CE

We previously demonstrated that in both mice and cynomolgus monkeys, the posterior intermediate mesoderm gives rise to KRT19^+^ CE, which, in turn, sequentially expresses GATA4 and NR5A1 to acquire the gonadal fate at E10 (embryonic day 10) in mice and E31 in cynomolgus monkeys (*8*). To precisely determine the specification timing of human gonads, we analyzed serial paraffin sections of human embryos at various stages (CS12, *n* = 1; CS13, *n* = 3; CS14, *n* = 1; CS15, *n* = 3; CS16, *n* = 2; CS17, *n* = 1; table S1). Similar to cynomolgus monkeys, a CS12 human embryo showed WT1^+^ posterior intermediate mesoderm extending ventrolaterally and forming KRT19^+^ early CE, which was demarcated from the FOXF1^+^ lateral plate mesoderm/splanchnic mesoderm (fig. S1, A to D). Regional differences in posterior intermediate mesoderm maturation along the anterior-posterior axis were observed; more anterior sections exhibited increased lateral extension of early CE suggestive of advanced maturation, whereas the posterior-most sections showed more immature morphology within the circumscribed mass of posterior intermediate mesoderm and no extensions of early CE (fig. S1D). These findings are consistent with previous studies on mice and cynomolgus monkey embryos and suggest that CE originates from posterior intermediate mesoderm (*8*).

We next sought to determine the onset of human gonadogenesis using GATA4 and NR5A1 as markers for the emerging gonads (*6*, *8*, *10*, *15*). GATA4 expression was not detected in CS12 to CS13 embryos (*n* = 4) (fig. S1, D to F). A weak GATA4 signal was first observed in a CS14 embryo within the WT1^+^NR5A1^−^ pseudo-stratified CE at the mid-posterior regions (figs. S1F and S2, A and B), which we termed gonadogenic CE. Early in 5 weeks postfertilization (wpf) (CS15, *n* = 3), WT1^+^GATA4^+^NR5A1^+^*LHX9*^+^ nascent gonad progenitors were observed at the ventrolateral aspect of the mesonephros as a pseudo-stratified region within KRT18/KRT19^+^ CE (fig. S2, A to D). NR5A1^+^ gonad progenitors were encompassed by the region expressing GATA4, which extended more broadly posteriorly and medially (fig. S2, C, E, and F). By late 5 wpf, the gonads became unequivocally recognizable by histology (CS16, *n* = 2) (fig. S2, A and B). These findings reveal that human gonadogenesis is initiated at CS14 to CS15 through the sequential activation of GATA4 and NR5A1 within the posterior CE, similar to mice and cynomolgus monkeys (*8*, *15*).

### Specification of the adrenal cortex in the adrenogenic CE in humans

**Fig. 1.**
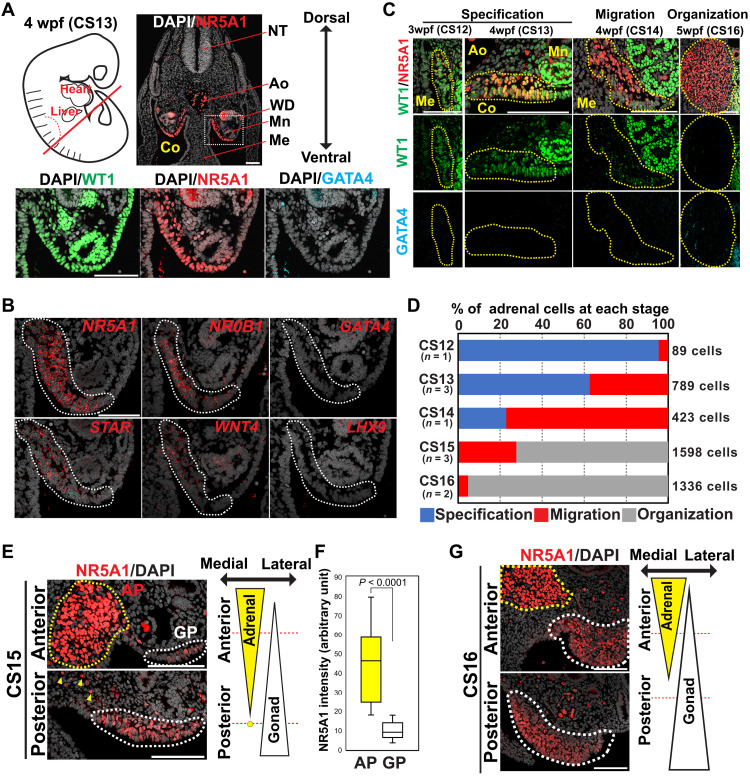
Specification of the adrenocortical lineage in humans. (**A**) Top left: Schematic of a human embryo at 4 weeks postfertilization (wpf) (CS13), with a red line indicating the approximate plane where the transverse sections were made for IF analysis. Top right: IF image of the embryo for NR5A1 (red) merged with 4merged with embryo for te p (DAPI) (white). Bottom: Magnified IF images of the region outlined by a dotted line for WT1 (green), NR5A1 (red), and GATA4 (cyan), merged with DAPI. Ao, aorta; Co, coelom; Me, mesentery; Mn, mesonephros; NT, neural tube; WD, Wolffian duct. Scale bar, 100 μm. (**B**) In situ hybridization (ISH) images of the coelomic angle (embryos, CS13) for the indicated markers (red) merged with DAPI (white). Dotted lines indicate the adrenogenic CE. Scale bar, 100 μm. (**C**) IF images of the coelomic angle at the indicated stages for WT1 (green), NR5A1 (red), and GATA4 (cyan). Merged images for WT1, NR5A1, and DAPI (white) are shown at the top. The yellow dotted line encircles the adrenogenic CE or adrenal primordium. Scale bars, 100 μm. (**D**) Percentage of adrenocortical cells at the indicated morphogenetic stages (specification, migration, and organization) for each embryonic stage (CS12 to CS16). Numbers of embryos and counted cells are shown. (**E**) IF images of the transverse sections taken from the indicated position from an embryo at CS15. Merged images of NR5A1 (red) and DAPI (white) are shown. The yellow and white dotted lines outline the adrenal primordium and gonad progenitor, respectively. The yellow arrowheads indicate several scattered cells of adrenal origin. Scale bars, 100 μm. (**F**) Box plot showing the distribution of NR5A1 intensities on the adrenal primordium (AP) and gonad progenitor (GP) cells as assessed by IF on the embryo used in (E). *P* < 0.0001 (Mann-Whitney test). (**G**) IF images of the transverse sections from an embryo at CS16. Scale bars, 100 μm.

During the immunofluorescence (IF) studies on emerging gonads, we incidentally found a WT1^+^GATA4^−^NR5A1^+^ region within the CE in 3 to 4 wpf embryos (CS12 to CS14) (Fig. 1, A and B, and fig. S3A). This region was seen only at the anterior portion of embryos (CS13, *n* = 2) (fig. S3B) and had markers of CE (KRT18^+^KRT19^+^CDH1^−^) (*8*) but was negative for a mesonephric marker, PAX2 (*16*), suggesting that it represents a portion of the CE (fig. S3C). Unexpectedly, the earliest stage embryo in which these cells were observed was at 3 wpf (CS12, *n* = 1), suggesting that the GATA4^−^NR5A1^+^ cells emerge shortly after the establishment of CE (Fig. 1C and fig. S3A). These CE cells gradually transitioned into the migration stage, as they increased NR5A1 expression and lost WT1 and KRT19 expression and eventually organized into the tightly packed adrenal primordium (organization stage) (5 wpf, CS16) (Fig. 1, C and D, and fig. S3, D and E) (*10*, *12*). Accordingly, these cells express key adrenocortical markers—*NR5A1*, *STAR*, *NR0B1*, and *WNT4*—but lack the gonadal markers—*GATA4* and *LHX9* (Fig. 1B). Thus, in humans, the adrenocortical lineage is first specified within a portion of the anterior CE, which we designate the adrenogenic CE. Moreover, the adrenocortical and gonadal lineages are specified independently, as gonadogenic CE did not appear until CS14 and was only observed in the posterior sections where very few GATA4^−^NR5A1^+^ adrenogenic CE cells were detected (fig. S4A). At CS15 to CS16 when gonad progenitors appear, the WT1^−^GATA4^−^NR5A1^bright+^ adrenal primordium had already formed an organized mass, medial to the mesonephros, and was separated from the WT1^+^GATA4^+^NR5A1^weak+^ emerging gonad progenitors (Fig. 1, E to G, and fig. S4, B and C). Cells surrounding the adrenogenic CE or adrenal primordium largely consisted of WT1^−^GATA4^−^NR5A1^−^ and WT1^+^GATA4^−^NR5A1^−^ cells, and cells indicative of transitioning into the gonadogenic CE or gonad progenitors (e.g., GATA4^+^NR5A1^+^, GATA4^+^WT1^+^, or WT1^+^NR5A1^+^ cells) were not observed, making the conversion of the adrenal lineage to the gonadal lineage less likely (fig. S4, A and B). Moreover, these two lineages exhibited a distinct distribution pattern along the anterior-posterior axis; the gonads extending more posteriorly than adrenal primordium (CS15, *n* = 3) (Fig. 1E and fig. S4B).

Previous studies in mice showed that germ cells required for eventual gamete production first migrate into the adrenogonadal primordium and redistribute into the gonads after segregation of the gonads and adrenal primordium (*7*). In humans, although primordial germ cells migrate into both the adrenal primordium and the gonads at early stages (4 to 5 wpf), they were selectively lost in the adrenal cortex thereafter (6 wpf). Thus, species-specific mechanisms of primordial germ cell migration, redistribution, and survival govern future gamete generation within gonads (fig. S4, D and E).

### Specification of the adrenal cortex in cynomolgus monkeys

**Fig. 2.**
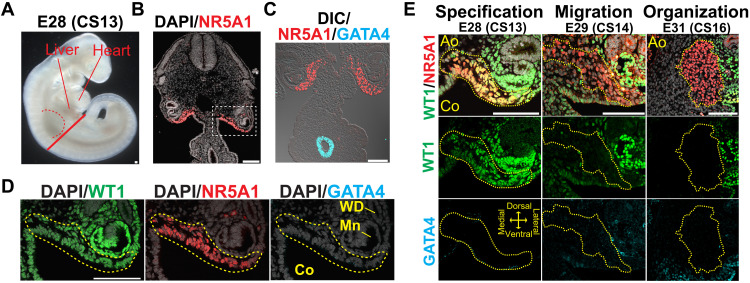
Specification of the adrenocortical lineage in cynomolgus monkeys. (**A**) Bright-field image of a cynomolgus embryo at E28 (CS13). The red dashed line denotes the forelimb bud. The solid red line indicates the approximate plane where the transverse sections were taken for IF analysis. (**B** and **C**) IF images of the section for NR5A1 (red) merged with DAPI (white) (B) or GATA4 (cyan), and differential interference contrast (DIC) image (white) (C). Scale bars, 100 μm. (**D**) Magnified images of the region outlined in (B) for WT1 (green), NR5A1 (red), and GATA4 (cyan), merged with DAPI (white). NR5A1^+^ adrenogenic CE are outlined by the yellow dashed line. Scale bar, 100 μm. (**E**) IF images of the coelomic angle of cynomolgus embryos at the indicated morphogenetic and embryonic stages for WT1 (green), NR5A1 (red), and GATA4 (cyan). Merged images for WT1, NR5A1, and DAPI (white) are shown. The yellow dotted lines outline the adrenogenic CE or adrenal primordium. Scale bars, 100 μm.

To evaluate whether the mode of adrenal specification is conserved among primates, we performed IF analyses on serial sections of cynomolgus monkey embryos (CS13 to C16, *n* = 11; table S1). WT1^+^GATA4^−^NR5A1^+^ adrenogenic CE first emerged at E28 (CS13) within the KRT18^+^KRT19^+^CDH1^−^PAX2^−^ CE (Fig. 2, A to D, and fig. S5, A and B). Disruption of basement membranes was noted along adrenogenic CE, suggestive of an active epithelial-to-mesenchymal transition (EMT)–like change (fig. S5B). Upon specification, adrenogenic CE underwent migration while gradually losing WT1 and KRT19 expression and formed a tightly organized WT1^−^GATA4^−^NR5A1^+^ adrenal primordium by E31 (CS16) (Fig. 2E and fig. S5, B to D). When WT1^+^GATA4^+^NR5A1^+^ gonad progenitors formed at E31, the adrenocortical cells were already organized into adrenal primordium, without spatially overlapping with the gonad (fig. S5E). These results suggest that in humans and cynomolgus monkeys, both adrenocortical and the gonadal lineages originate from the CE, albeit in a spatially and temporally distinct manner.

### Transcriptomic changes accompanying adrenogenic CE specification in humans

We next sought to understand the molecular events associated with the segregation and subsequent development of adrenocortical and gonadal cell lineages using single-cell transcriptomics. After conducting quality control analyses (fig. S6A), we first explored adrenocortical development immediately after the specification of adrenogenic CE. Analyzing whole urogenital ridge at 4 wpf (CS14) by the uniform manifold approximation and projection (UMAP) revealed *WT1*^+^*KRT18*^+^*KRT19*^+^ CE, portions of which showed *GATA4* or *NR5A1* expression in a mutually exclusive manner (fig. S6B). Various other cell types were also annotated on the basis of known marker genes and differentially expressed genes (DEGs; fig. S6, C and D, and table S2).

**Fig. 3.**
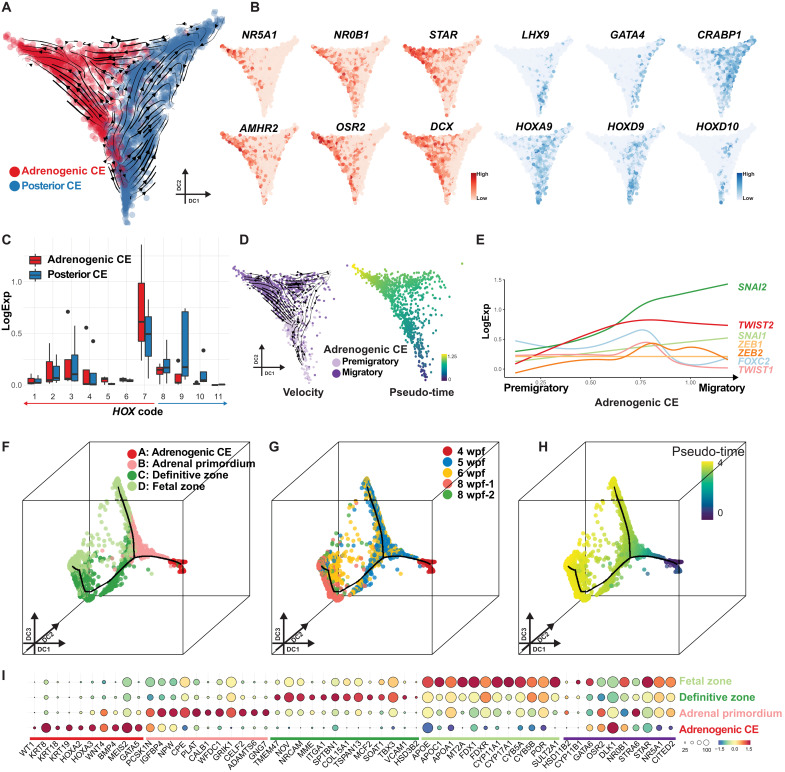
Transcriptomic dynamics accompanying adrenal specification and maturation in humans. (**A**) Single-cell transcriptomes of the urogenital ridge at 4 wpf, projected on diffusion map with overlaid RNA velocity. Cells are colored according to cell cluster (red, adrenogenic CE; blue, posterior CE). Black arrows show the velocity field. DC, diffusion component. (**B**) Expression of key markers of adrenogenic CE (red) and posterior CE (blue). (**C**) Summary of *HOX* gene expression in adrenogenic CE and posterior CE. Log-normalized expression of *HOX* genes belonging to the same paralogs is summarized together. Numbers 1 to 7 indicate anterior *HOX* genes, and numbers 8 to 11 indicate posterior *HOX* genes. *HOX12* and *HOX13* are filtered out as outliers because of extremely low expression. (**D**) Left: Subclustering of adrenogenic CE into two cell clusters, premigratory and migratory adrenogenic CE, with overlaid RNA velocity. Right: Pseudo-time is projected on the same plots. (**E**) Expression dynamics of key transcription factors associated with EMT, aligned along pseudo-time as in (D). (**F** to **H**) Reclustering of the adrenocortical lineage (defined in fig. S8C) and projection of four clusters onto the diffusion map. Cells are colored according to cell cluster (F), sample origin (G), or pseudo-time (H). (**I**) Key marker gene expression. Putative genes involved in adrenal specification and steroidogenesis are also shown at the far right, underlined by a purple bar.

Reclustering of the CE cluster revealed two subclusters. The first cluster (colored in red) was annotated as adrenogenic CE given its characteristic gene expression pattern (*GATA4*^−^*LHX9*^−^*NR0B1*^+^*STAR*^+^*NR5A1*^+^) (Figs. 1, A and B, and 3, A and B). The second cluster (colored in blue) expressed posterior *HOX* genes (e.g., *HOXA9* and *HOXD9*), encompassed *GATA4*^+^*LHX9*^+^*NR5A1*^−^ gonadogenic CE, and was tentatively annotated as posterior CE (Fig. 3, A to C, and fig. S6E). Notably, the adrenogenic CE cluster could be further divided into two subclusters (Fig. 3D and fig. S7A). RNA velocity and pseudo-time trajectory analyses revealed overall directional lineage progression from one cluster (bottom) to the other (top) (Fig. 3D). These clusters differed in expression of *NR5A1* and *WT1*, reflecting the immunophenotypic differences between NR5A1^low^WT1^high^ premigratory adrenogenic CE and NR5A1^high^WT1^low^ migratory adrenogenic CE (fig. S7, B and C). Accordingly, genes related to cell migration were up-regulated in migratory adrenogenic CE (fig. S7, B and D, and table S3). Genes enriched in Gene Ontology (GO) terms such as “adrenal gland development” and “cholesterol metabolic process” were up-regulated along the pseudo-time, suggesting initiation of expression of the adrenal steroidogenic machinery (fig. S7D). Moreover, migratory adrenogenic CE showed up-regulation of many genes related to “extracellular matrix” and key transcription factors critical for EMT (e.g., *SNAI1*, *SNAI2*, *TWIST2*, and *ZEB2*) (Fig. 3E; fig. S7, B and D; and table S3), suggesting that the EMT-like morphogenetic changes are operative during the transition (*17*). While adrenogenic CE expressed some of the previously identified mouse adrenal specifier genes such as *PKNOX1* (*PREP*) and *WT1*, other key specifiers such as *CITED2*, *PBX1*, or *HOXB9* were only rarely and weakly expressed at this stage (*4*, *9*), suggesting a divergence of the adrenal specification genetic pathway or developmental timeline between mice and humans (fig. S7E).

### Gene expression dynamics during maturation of adrenocortical cells

We next sought to understand the dynamics of adrenocortical cell maturation after specification. UMAP plot of whole adrenals at 5 to 8 wpf and the urogenital ridge at 4 wpf revealed *NR5A1*^+^*STAR*^+^ fetal adrenocortical lineage (adrenogenic CE, cluster 3; adrenal cortex, cluster 8) (fig. S8, A to C). Other lineages previously observed in fetal adrenals were also identified on the basis of the expression of marker genes (fig. S8, C and D, and table S4). Reclustering of isolated adrenocortical lineages and projection onto the diffusion map revealed a lineage trajectory in which four clusters (clusters A to D) aligned along pseudo-time, which were also concordant with sample origins (Fig. 3, F to H, fig. S9A, and table S5). Cluster A was annotated as adrenogenic CE, as it expressed markers of previously defined adrenogenic CE (e.g., *WT1*, *KRT19*, and *NR5A1*) (Fig. 3, F and I). As the lineage progressed toward cluster B, cells up-regulated some steroidogenic genes (e.g., *NR5A1*, *APOA1*, and *FDXR*), while they down-regulated CE markers (e.g., *WT1*, *KRT19*, and *KRT18*) (Fig. 3, F to I, and fig. S9, B to D). Genes involved in early development and axial specification were also down-regulated (fig. S9, B to D). Notably, this cluster had yet to significantly up-regulate genes characteristic of the fetal zone (e.g., *CYP17A1* and *SULT2A1*) or the definitive zone (e.g., *MME*, *HSD3B2*, and *NOV*) (fig. S9D) and was therefore annotated as adrenal primordium. The lineage subsequently bifurcated into two branches (Fig. 3, F to H). In the first branch (branch 1, direct path), adrenal primordium directly fed into cluster D, which consisted predominantly of 6 to 8 wpf adrenals, highly expressing key enzymes required for the biosynthesis of adrenal androgens (e.g., *CYP17A1* and *SULT2A1*), feature characteristic of fetal zone adrenal cells (Fig. 3, F to I; and figs. S9, B to F, and S10, A to D) (*2*). The second branch (branch 2, indirect path) showed transition of adrenal primordium into the fetal zone through cluster C, herein annotated as definitive zone adrenal cells based on expression of known markers of the definitive zone (e.g., *NOV* and *HSD3B2*) (Fig. 3, F to I; and figs. S9, B to D and F, and S10, C and D) (*2*, *18*–*20*). Differential gene expression analysis showed a number of previously unidentified surface markers (*MME*, *ITGA1*, *NRCAM*, and *TMEM47*), which might be useful for the isolation and characterization of the definitive zone (Fig. 3I, figs. S9D and S10C, and table S5). We also noted that the definitive zone expressed various transcription factors/homeodomain proteins (e.g., *LEF1*, *ETV5*, *TBX3*, and *HOPX*), which might play roles in maintaining the stemness of the definitive zone (Fig. 3I and fig. S9B). Notably, fetal zone development through either the direct or indirect pathway was associated with up-regulation of genes required for steroid biosynthesis, while those associated with cell proliferation were down-regulated, consistent with the notion that the fetal zone contains terminally differentiated steroidogenic cells (figs. S9, C and E, and S10D). In contrast, the definitive zone exhibited overall lower expression of genes involving in steroid biosynthesis than the fetal zone, except *HSD3B2*, which was expressed higher in the definitive zone (fig. S10D), suggesting that the definitive zone may involve in Δ4 steroid biosynthesis (*18*, *20*). When fetal zone cells in cluster D were further separated into two clusters based on the trajectories (branches 1 and 2), fetal zone cells in branch 2 have higher expression of definitive zone markers (fig. S9E). Moreover, by projecting expression of the fetal and definitive zone markers simultaneously on the diffusion map, we found that cells expressing both markers were more frequently seen in fetal zone cells belonging to branch 2, further suggesting that they were derived from the definitive zone (fig. S9F). Together, our findings suggest that early adrenocortical development accompanies the gradual acquisition of steroidogenic function following direct or indirect development from a common progenitor, the adrenal primordium.

Recent studies in mice suggest that a functional interaction between the adrenal capsule and the fetal adrenal cortex maintains functional zonation through signaling mediated by the Hedgehog and WNT pathways (*14*). Consistent with this notion, we found that Hedgehog ligands are expressed in the adrenal cortex, while their receptors and downstream genes are expressed in the capsule (fig. S11, A to E). Notably, *RSPO3* (activator of canonical WNT pathway) was predominantly expressed in the capsule, whereas *WNT4* was expressed in both the capsule and cortex (fig. S11, B to E). *Wnt4* is predominantly expressed in the cortex in mice (*14*), which may suggest the divergence between mice and humans. These findings suggest interactions between the capsule and cortex that may play a role in maintaining the functional zonation of the human fetal adrenals.

### Adrenocortical and gonadal lineages bear distinct HOX codes

**Fig. 4.**
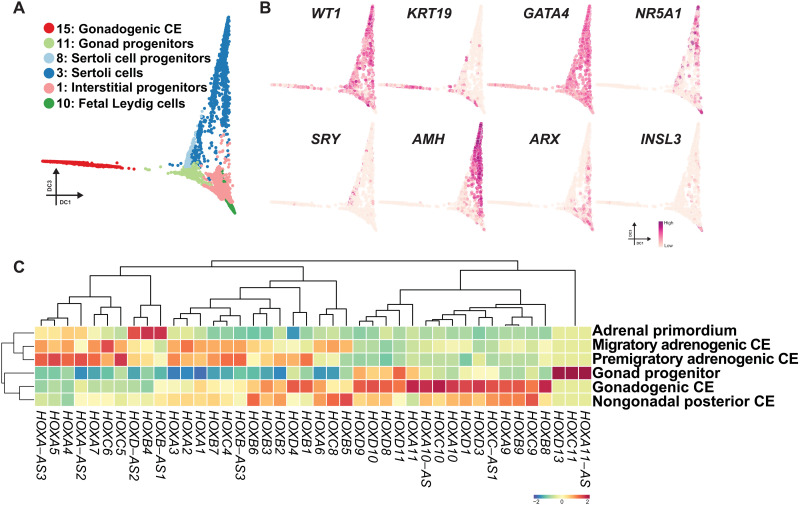
Transcriptomic dynamics during the maturation of adrenocortical cells. (**A**) Diffusion map showing the trajectory during human gonad development. Cells are colored according to cell cluster as in fig. S12C. (**B**) Key marker genes used for annotation of cell types projected on the diffusion map in (A). (**C**) Averaged expression values of *HOX* genes in the indicated cell clusters. *HOX* gene expression is *z* score normalized by each column, and cell clusters and genes are hierarchically clustered.

We next set out to explore the developmental trajectory of the early testicular lineages. To this end, clusters representing gonadal somatic cells were isolated from single-cell transcriptomes of the whole testes at 5 to 12 wpf and the urogenital ridge at 4 wpf and projected onto a diffusion map (Fig. 4, A and B; figs. S12, A to E, and S13, A to C; and table S6). These analyses revealed the lineage trajectory where *KRT19*^+^*WT1*^+^*GATA4*^+^*NR5A1*^−^ gonadogenic CE (cluster 15, predominantly 4 wpf) first progressed into *KRT19*^+^*WT1*^+^*GATA4*^+^*NR5A1*^+^ gonad progenitors (cluster 11, predominantly 5 wpf), which subsequently bifurcate into two lineages: *SRY*^+^ Sertoli cell progenitors (cluster 8)/*AMH*^+^ Sertoli cells (cluster 3) and *ARX*^+^ interstitial progenitor (cluster 1)/*INSL3*^+^ fetal Leydig cell lineages (cluster 10) (Fig. 4, A and B, and figs. 12E and 13, A to G).

Our transcriptome analysis and annotation of cell types present in the early adrenocortical or gonadal lineages allowed us to interrogate the potential relationship between these ontologically related lineages (fig. 13, H to J). Pairwise correlation analyses of cell types present in these two lineages showed an overall high degree of transcriptomic correlation (fig. 13I). Unbiased hierarchical clustering revealed gonad progenitors clustered with gonadogenic CE rather than adrenogenic CE (migratory and premigratory), further lending support to their distinct origins (fig. 13I). Moreover, these combined analyses reveal that adrenocortical and gonadal lineages exhibit distinct expression patterns of *HOX* genes; adrenocortical lineages expressed predominantly anterior *HOX* gene paralogs (*HOX1* to *HOX7*), whereas gonadal lineages had high levels of posterior *HOX* genes (*HOX8* to *HOX13*) with only a modest expression of anterior *HOX* genes (Fig. 4C and fig. S13, I to K). Together, these findings further support that the conclusion that adrenal and gonadal lineages have distinct origins characterized by differential expression of anterior versus posterior *HOX* codes in their early progenitors, respectively.

## DISCUSSION

**Fig. 5.**
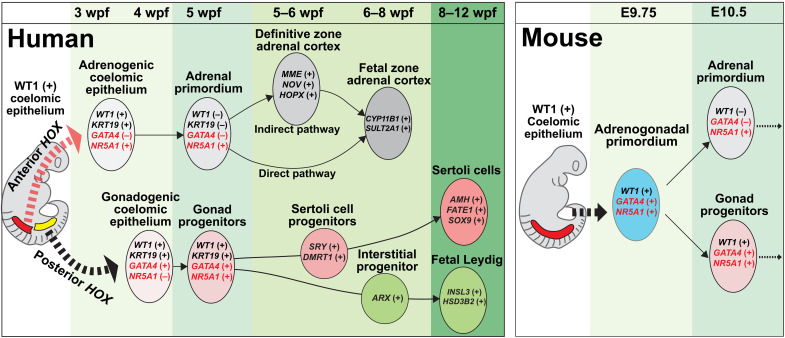
A model of adrenal and gonadal development in humans and mice. Schematic representation of a model of adrenal and gonadal development in humans (left) and mice (right).

In this study, we provide evidence that, in contrast to mice, the adrenal cortex in humans and cynomolgus monkeys originates in a spatially, temporally, and phenotypically distinct manner from that of the gonads (Fig. 5). Specifically, through histologic and transcriptomic analyses of early human gonadogenesis, we found that the gonad is established from the posterior CE at 4 to 5 wpf through the sequential activation of GATA4 (4 wpf, CS14) and NR5A1 (5 wpf, CS15), similar to mice and cynomolgus monkeys (Fig. 5 and fig. S1) (*8*). A previous single-cell RNA sequencing (scRNA-seq) study suggested that NR5A1 is not activated in the early gonad and GATA4 only appears at 6 to 7 wpf when testicular cord formation is initiated (*21*). However, this discrepancy may be explained by either differences in sensitivity of detection and/or potential differences in allocation of the fetal ages relative to our study, in which the onset of gonadogenesis was more in accord with the previous IF/histologic studies on human and monkey embryos (*8*, *10*, *11*). Serendipitously, during this analysis, we found that the adrenocortical lineage in humans is first specified within a portion of the CE that is more anterior to the gonadogenic CE. Moreover, gonadogenic CE appeared later in development than did adrenogenic CE and was only observed in the posterior sections where adrenogenic CE was not detected. Thus, our findings reveal that, in contrast to mice, the adrenocortical and gonadal lineages are specified independently in humans.

The origin of the fetal and definitive zones in the human fetal adrenal cortex has long been a source of controversy (*2*, *13*, *22*, *23*). Our lineage trajectory analyses of human adrenal cortex transcriptomes suggest the presence of a shared progenitor of the fetal and definitive zones (i.e., adrenal primordium), consistent with lineage tracing studies in mice (*5*). However, our trajectory analysis also revealed that the definitive zone in humans can further differentiate into the fetal zone with similar steroidogenic characteristics to those derived from the direct pathways. This finding is consistent with a previous model in which the fetal zone is derived from the definitive zone through continuous centripetal migration of definitive zone cells (migration theory) (*2*). What then determines the fate decision between the fetal zone and definitive zone? Our transcriptomic comparison showed the up-regulation of a number of transcription factors/cofactors specific to the definitive zone (fig. S9B). Among them, HOPX is a key transcription cofactor, which is known to play key role in maintaining the stemness in neuronal or intestinal stem cells (*24*, *25*) and known to down-regulate upon differentiation of the definitive zone by trophic stimuli mediated by adrenocorticotropic hormone (ACTH) (*26*). Moreover, capsular cells, whose transcriptomes are also illustrated in this study, are continuously juxtaposed with the definitive zone during fetal life (*2*). We found that human capsular cells are *RSPO3*^+^ similar to mice (*27*), which might serve as a niche signaling to maintain the definitive zone fate. These possibilities warrant further investigation. Our unifying model of early adrenal development will therefore shed new insight into the molecular ontogeny of functional zonation in human adrenal glands (Fig. 5).

We found that the adrenocortical and gonadal lineages exhibit different expression patterns of *HOX* genes, which confer regional identity along anterior-posterior axis through a “*HOX* code” that results from timed activation of *HOX* paralogous genes from the 3′ to 5′ direction as nascent mesodermal cells emigrate from the posterior growth zone (*28*, *29*). While the adrenals preferentially express anterior *HOX* genes (i.e., *HOX1* to *HOX7*), the gonads express more posterior *HOX* genes (i.e., *HOX8* to *HOX13*) (Fig. 4C and fig. S13K). Thus, by the time the adrenals and the gonads are specified within the anterior and posterior CE, respectively, a combination of their distinct regional identity conferred by the HOX code combined with inductive signals likely establishes the unique transcriptional regulatory modules that direct adrenal or gonad-specific cell fates. In support, the HOX/PBX1/PREP1 complex appears to be critical for the initiation of adrenal development upon segregation from the adrenogonadal primordium in mice (*4*). Thus, our findings suggest that distinct induction of human adrenal and gonadal fate in vitro might be achieved through modulating signals for anterior-posterior regionalization (e.g., canonical WNT signaling) as exemplified by the recent success in the induction of anteriorly derived ureteric bud and posteriorly derived metanephric mesenchyme (*30*, *31*).

In summary, our findings reveal the distinct origin of the human and infrahuman primate adrenal cortex and gonads and provide an example of the divergence of organ morphogenesis between species. Note that although our multimodal analyses thoroughly addressed the developmental trajectories of human adrenocortical and gonadal lineages, the rigorous characterization of lineage relationships requires genetic lineage tracing or live imaging. It is also of great interest to functionally interrogate the differential requirement of WT1, GATA4, or other transcription factors in human adrenocortical and gonadal specification (*4*, *6*, *9*, *32*, *33*). However, these experiments are not possible in humans due to obvious bioethical reasons. Infrahuman primates or stem cell–based in vitro models might be used as a surrogate to address these key questions in the future. The molecular details of early lineage diversification of human and cynomolgus monkey adrenals and gonads illustrated in this study will serve as both a framework for understanding the molecular pathology of human disorders (e.g., disorders of sex development and primary adrenal insufficiency) and also provide essential insight into the future reconstitution of these lineages in vitro from pluripotent stem cells to advance disease modeling and regenerative medicine.

## MATERIALS AND METHODS

### Collection of human embryo samples

Urogenital organs at 3 to 8 wpf were obtained from donors who had provided informed consent and underwent elective abortion at the University of Pennsylvania, Ookouchi Obstetrics and Gynecology Clinic, and Fukuzumi Obstetrics and Gynecology Clinic. Embryo ages were determined through ultrasonographic measurement of the crown-rump length. All experimental procedures were approved by the Institutional Review Boards at the University of Pennsylvania (#832470) and the Hokkaido University (19-066). In most embryos, the sex was determined with sex-specific polymerase chain reaction (PCR) performed on genomic DNA isolated from trunk tissues with primers specific to the *ZFX*/*ZFY* loci (*8*, *34*). Embryos were dissected in RPMI 1640 (Roche). Human embryos used in this study are listed in table S1.

### Collection of cynomolgus monkey embryo samples

All procedures in cynomolgus monkeys were approved by the Institutional Animal Care and Use Committee of the Shiga University of Medical Science. The assisted reproductive technologies in cynomolgus monkeys, including oocyte collection, intracytoplasmic sperm injection, preimplantation embryo culture, and transfer of preimplantation embryos into recipient mothers, were as previously reported (*35*). The light cycle comprised 12 hours of artificial light from 8 a.m. to 8 p.m. Water was made available ad libitum. The temperature and humidity in the animal rooms were maintained at 23° to 27°C and 45 to 55%, respectively. Implanted embryos were scanned with transabdominal ultrasound and recovered by cesarean section under full anesthesia. A total of 10 embryos were harvested, ranging in embryonic age from E28 to E32 (CS13 to CS16 embryos; *n* = 6 male, 3 female, and 1 unknown). We included samples obtained in previous studies in the analysis (*8*, *34*). The recipient females were maintained after surgery. The sex of each sampled fetus was determined by sex-specific PCR with primers targeting the ZFX/ZFY loci (*8*, *34*). Cynomolgus monkey embryos used in this study are listed in table S1.

### Antibodies

The primary antibodies used in this study included goat anti-FOXF1 (R&D Systems, AF4798; RRID:AB_2105588), goat anti-GATA4 (Santa Cruz Biotechnology, sc-1237; RRID:AB_2108747), mouse anti-GATA4 (Santa Cruz Biotechnology, sc-25310; RRID:AB_2108747), mouse anti-MME (Biocare Medical, CM129; RRID:AB_10579027), mouse anti-NR5A1 (Novus Biologicals, N1665; RRID:AB_1962633), mouse anti-TFAP2C (Santa Cruz Biotechnology, sc-12762; RRID:AB_667770), rabbit anti-CDH1 (Cell Signaling Technology, 3195S; RRID:AB_2291471), rabbit anti-laminin (Abcam, ab11575; RRID:AB_298179), rabbit anti-WT1 (Abcam, ab89901; RRID:AB_2043201), rabbit anti-KRT18 (Abcam, ab133263; RRID:AB_11155892), rabbit anti-KRT19 (Abcam, ab52625; RRID:AB_2281020), and rabbit anti-PAX2 (BioLegend, 901002; RRID:AB_2734656). The secondary antibodies included Alexa Fluor 488–conjugated donkey anti-rabbit immunoglobulin G (IgG) (Life Technologies, A21206; RRID:AB_2535792), Alexa Fluor 488–conjugated donkey anti-mouse IgG (Life Technologies, A32766; RRID:AB_2762823), Alexa Fluor 568–conjugated donkey anti-mouse IgG (Life Technologies, A10037; RRID:AB_2534013), Alexa Fluor 568–conjugated donkey anti-rabbit IgG (Life Technologies, A10042; RRID:AB_2534017), Alexa Fluor 647–conjugated donkey anti-goat IgG (Life Technologies, A21447; RRID:AB_2535864), and Alexa Fluor 647–conjugated donkey anti-rabbit IgG (Life Technologies, A31573; RRID:AB_2536183).

### IF analyses on paraffin sections

IF analyses for human and cynomolgus monkey embryos were performed on paraffin sections. Anterior tissues (the head and the upper chest above the level of the heart) were removed before fixation to increase the perfusion with 10% neutral buffered formalin. Samples were kept in ice-cold RPMI 1640 medium during dissection and placed in formalin fixative within 1 hour (cynomolgus embryos) or 2 hours (human embryos) after surgical recovery of the embryos. Samples submerged in formalin were incubated ~24 hours at room temperature with gentle rocking. After dehydration, tissues were embedded in paraffin, serially sectioned at 4 mm in thickness with a microtome, and placed on glass slides (Platinum Pro). Embryos were oriented perpendicularly to the surface of the mold to obtain transverse sections. For one embryo at 8 wpf, the adrenal gland was isolated and fixed in 10% neutral formalin before being embedded in paraffin, oriented perpendicularly to the surface of the mold. The embryos embedded in paraffin blocks were serially sectioned at 4 μm in thickness with a microtome (Leica RM2035) and placed on microscopic glass slides (Superfrost Plus, Thermo Fisher Scientific). Paraffin sections were then deparaffinized with xylene. Antigens were retrieved by treatment of sections with HistoVT One (Nacalai Tesque) for 35 min at 90°C and then for 10 min at room temperature. The slides were washed twice with phosphate-buffered saline (PBS) and then incubated with blocking solution (5% donkey serum, 0.2% Tween 20, and 1× PBS) for 1 hour at room temperature. The primary antibody incubation was performed overnight at 4°C, and slides were washed six times with PBS (20 min each) and then incubated with secondary antibodies in blocking solution and 4′,6-diamidino-2-phenylindole (DAPI; 1 μg/ml) for 50 min. Slides were subsequently washed six times with PBS before being mounted in VECTASHIELD mounting medium (Vector Laboratories) for confocal microscopy analysis (Leica, SP5-FLIM inverted). Confocal images were processed in Leica LAS X (version 3.7.2).

### In situ hybridization on paraffin sections

In situ hybridization (ISH) on formalin-fixed paraffin-embedded sections was performed with the ViewRNA ISH Tissue Assay Kit (Thermo Fisher Scientific) with gene-specific probe sets for human *NR5A1*, *LHX9*, *GATA4*, *WNT4*, *STAR*, *NR0B1*, *NOV*, *RSPO3*, and *PDGFRA*. Experiments were conducted according to the manufacturer’s instructions (incubation with pretreatment buffer for 12 min, protease treatment for 6 min 30 s, and use of FastRed as a chromogen). Slides were counterstained with DAPI (1 μg/ml) for 50 min before being mounted in VECTASHIELD mounting medium for confocal microscopic analysis.

### Staging of embryos and histologic quantification of adrenals, gonads, and germ cells

Embryos were staged according to the CS whenever possible (*36*). All image analyses were performed in ImageJ. For quantification of adrenocortical cells, sections containing NR5A1^+^GATA4^−^ adrenogenic CE/adrenal primordium were first determined with IF conducted on every five sections (~20-μm interval). Then, the regions containing adrenogenic CE/adrenal primordium were partitioned into quadrants, and at least one section per quadrant was analyzed to determine the number of cells. Cells in specification stage were defined as NR5A1^+^GATA4^−^ cells within the CE consisting of a three–cell layer thickness. Cells at the migration stage were defined as NR5A1^+^GATA4^−^ cells not in the CE and not forming a cluster of a size meeting the criteria for the organization stage. Cells at the organization stage were defined as NR5A1^+^GATA4^−^ cells forming >20 coherent cell clusters (<4-μm distance between cells). Cells surrounding NR5A1^+^GATA4^−^ adrenal cells (<4-μm distance) in CS14 and CS15 embryo sections [used in fig. S4 (A and B)] were evaluated for immunoreactivity for WT1, GATA4, or NR5A1 by ImageJ.

### 10x Genomics scRNA-seq library preparation

Fetal urogenital organs at 4 to 12 wpf [four adrenals (5 wpf, *n* = 1; 6 wpf, *n* = 1; 8 wpf, *n* = 2), six testes (5 wpf, *n* = 2; 6 wpf, *n* = 1; 8 wpf, *n* = 2; 12 wpf, *n* = 1), and one whole urogenital ridge (4 wpf, *n* = 1)] were used for scRNA-seq with a Chromium Single Cell 3′ Reagent Kit (v3 chemistry). For preparation of 4 wpf embryos, the lateral abdominal wall and limb bud were first trimmed with forceps and ophthalmic scissors, and then the entire urogenital ridge containing the CE, mesonephros, and a portion of the proximal mesentery was isolated for downstream processing. For the 5 to 8 wpf samples, under a dissection microscope, the entire adrenal glands and/or testes were dissected by removal of the surrounding fibroadipose tissues. The 5 wpf samples were small and partly disrupted during the clinical procedure; therefore, complete separation of the gonads or adrenals from the surrounding tissues could not be performed with certainty. Thus, a small portion of the surrounding tissues remained attached when samples were processed for downstream assays.

Following procedures previously described (*8*), we isolated embryonic fragments, washed twice with PBS, then minced with ophthalmic scissors and fine forceps in 500 μl of 0.1% trypsin/EDTA solution, and then incubated for 9 min at 37°C with gentle pipetting every 3 min. After quenching of the reaction by addition of 500 μl of Dulbecco’s modified Eagle’s medium supplemented with 10% fetal bovine serum, cell suspensions were strained through a 70-μm nylon cell strainer and centrifuged for 220*g* for 5 min. Cell pellets were subsequently resuspended in 0.1% bovine serum albumin in PBS and counted for the number of cells. All samples were stained with trypan blue and confirmed to be >80% viable. Cells were loaded into Chromium microfluidic chips and used to generate single-cell gel bead emulsions with a Chromium controller (10x Genomics) according to the manufacturer’s protocol. Gel bead emulsion–reverse transcription was performed with a C1000 Touch Thermal Cycler equipped with a deep-well head (Bio-Rad). All subsequent cDNA amplification and library construction steps were performed according to the manufacturer’s protocol. Libraries were sequenced with a NextSeq 500/500 high output kit v2 (150 cycles) (FC-404-2002) on an Illumina NextSeq 550 sequencer.

### Mapping reads of 10x Chromium scRNA-seq and data analysis

Raw data were demultiplexed with the mkfastq command in Cell Ranger (v2.1.0) to generate FASTQ files. Trimmed sequence files were mapped to the reference genome for humans (GRCh38) provided by 10x Genomics. Read counts were obtained from outputs from Cell Ranger.

Secondary data analyses were performed in Python with Scanpy 1.8.1 or in R (v.3.6.1) with the Seurat (v.4.0), ggplot2 (v.3.3.2), gplots (v.3.0.3), qvalue (v.2.18.0), maptools (v.0.9-9), genefilter (v.1.68.0), rgl (v.0.100.54), dplyr (v.0.8.3), and matrix (v.1.2-18) packages and Excel (Microsoft). Unique molecular identifier (UMI) count tables were first loaded into R by using the Read10x function, and Seurat objects were built from each sample. Cells with fewer than 200 genes, an aberrantly high gene count above 7000, or a percentage of total mitochondrial genes of >15% were filtered out. Of the ~89,477 cells for which transcriptomes were available, 72,257 cells passed quality control dataset filters and were used in downstream analysis. We detected ~3616 median genes per cell at a mean sequencing depth of ~60,509 reads per cell (fig. S6A). Samples were combined, and the effects of mitochondrial genes, cell cycle genes, and batches were regressed out with SCTransform during normalization in Seurat and then converted to log_2_ (CP10M + 1) values. Mitochondrial genes and cell cycle genes were excluded during cell clustering, dimensional reduction, and trajectory analysis. Cells were clustered according to a shared nearest neighbor modularity optimization–based clustering algorithm in Seurat. Clusters were annotated on the basis of previously characterized marker gene expression with the FeaturePlot function and the gene expression matrix file, and cluster annotation was generated for downstream analyses. Dimensional reduction was performed with the top 3000 highly variable genes and the first 30 principal components (PCs) with Seurat. DEGs in different clusters were calculated with Seurat findallmarkers, with thresholds of an average log_2_ fold change (FC) of approximately 0.25 and *P* < 0.01. Developmental trajectories of cells were simulated with the first 30 PCs and 30 diffusion components by DiffusionMap in destiny and Scanpy. Trajectory principal lines were fitted with ElPiGraph.R. Pseudo-time was calculated with diffusion pseudo time (dpt) in destiny. RNA velocity matrices were generated with velocyto 0.17 and then analyzed with scVelo 0.25. DEGs between two groups in scatterplots were identified with edgeR 3.34.1 through application of a quasi-likelihood approach and the fraction of detected genes per cell as covariates. The DEGs were defined as those with false discovery rate (FDR) < 0.01, *P* < 0.01, and log_2_ FC > 1. The cell cycle was analyzed with CellCycleScoring in Seurat. Data were visualized with ggplot2 and pheatmap. Genes in the heatmap were hierarchically clustered according to Euclidean distance, scaled by row, and then visualized with pheatmap. HOX code scoring was calculated with area under the curve methods in AUCell 1.14.0. GO enrichment was analyzed with DAVID v6.8. Cluster D in fig. S9A was defined on the basis of clustering in Seurat with a resolution of 0.6. With increased resolution parameters, we could identify subclusters within cluster D, which, however, did not yield subclusters that fitted well with the two paths (direct or indirect path) defined by the diffusion map. Thus, we used ElPiGraph.R to fit principal curves to the data. After fitting, the fetal zone cells (cluster D) were assigned to either branch 1 (855 cells) or branch 2 (390 cells). Nineteen cells were assigned to neither trajectories due to the same distances to two trajectories. The DEGs were defined as those with FDR < 0.01, *P* < 0.01, and log_2_ FC > 1. The whole process was same as other pairwise comparisons in this study.

## Notes

View/request a protocol for this paper from *Bio-protocol*.
