# Supplementary material for: The developmental origin and the specification of the adrenal cortex in humans and cynomolgus monkeys
Source: Sci Adv. 2022 Apr 20;8(16):eabn8485. doi: 10.1126/sciadv.abn8485 (PMC9020778; doi:10.1126/sciadv.abn8485)
Supplement: Supplementary file 1 — Figs. S1 to S13 [file sciadv.abn8485_sm.pdf]

Science Advances
AAAS

Supplementary Materials for

The developmental origin and the specification of the adrenal cortex in humans and cynomolgus monkeys

Keren Cheng, Yasunari Seita, Taku Moriwaki, Kiwamu Noshiro, Yuka Sakata, Young Sun Hwang, Toshihiko Torigoe, Mitinori Saitou, Hideaki Tsuchiya, Chizuru Iwatani, Masayoshi Hosaka, Toshihiro Ohkouchi, Hidemichi Watari, Takeshi Umazume, Kotaro Sasaki*

*Corresponding author. Email: ksasaki@upenn.edu

Published 20 April 2022, *Sci. Adv.* **8**, eabn8485 (2022)
DOI: 10.1126/sciadv.abn8485

The PDF file includes:

Legends for tables S1 to S6
Figs. S1 to S13

Other Supplementary Material for this manuscript includes the following:

Tables S1 to S6

Supplementary Tables

Table S1. Human and cynomolgus embryos used in this study.

Table S2. Differentially expressed genes across cell clusters in the human urogenital ridge at 4 wpf.

Table S3. Differentially expressed genes between the pre-migratory and migratory adrenogenic coelomic epithelium.

Table S4. Differentially expressed genes across cell clusters present in human adrenal glands (5-8 wpf) and the urogenital ridge (4 wpf).

Table S5. Differentially expressed genes across cell clusters in human adrenal lineages.

Table S6. Differentially expressed genes across cell clusters in human testes and the urogenital ridge (4 wpf).

Supplementary Figure 1, Cheng et al.

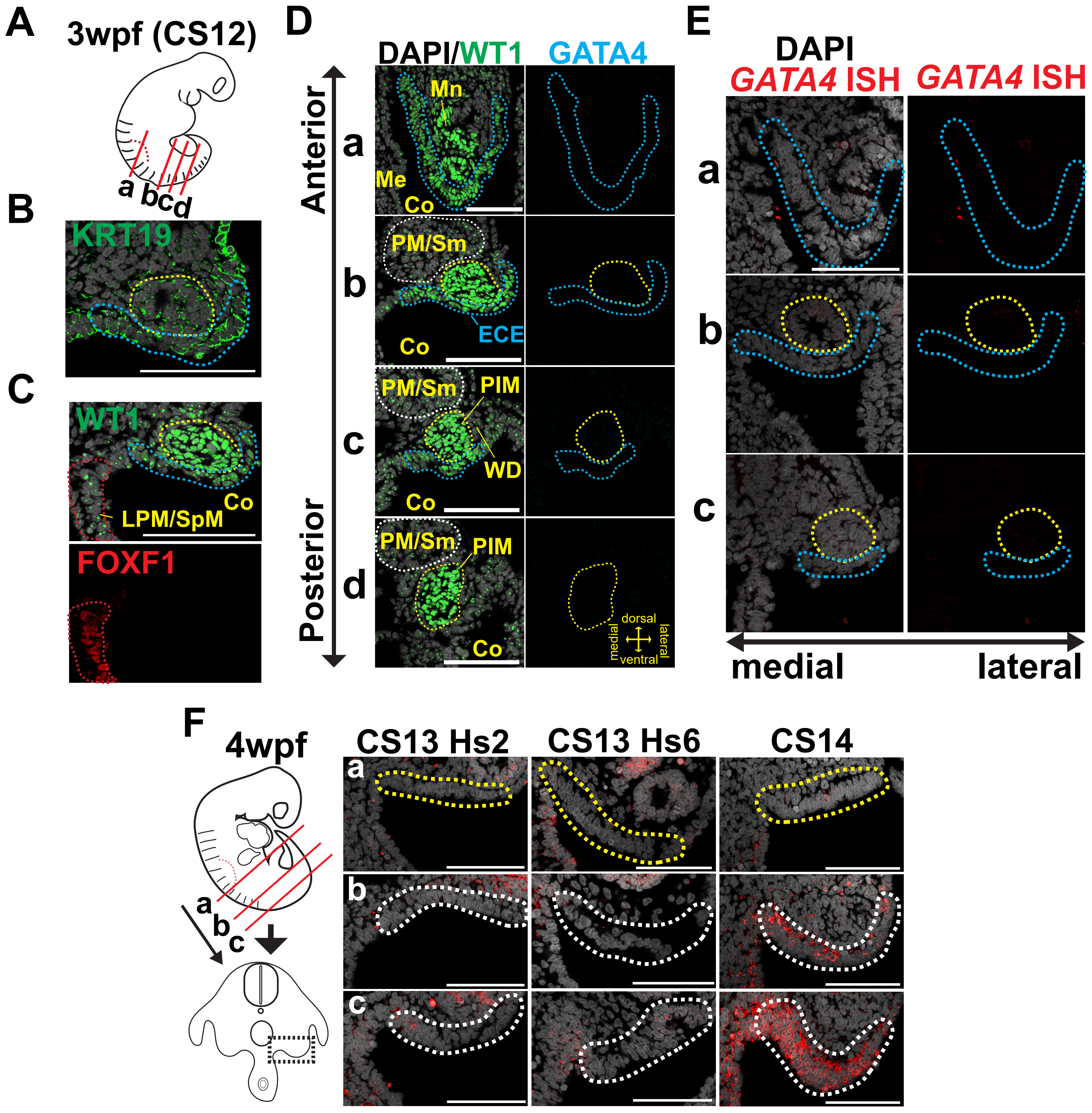

Fig. S1. Specification of coelomic epithelium in humans.

(**A**) Schematic of a human embryo at 3 wpf (CS12), with red lines indicating the approximate planes where the sections were taken in (**B–E**).

(**B**) IF image of a transverse section taken at level b of the embryo in (**A**) for KRT19 (green) merged with DAPI (white). Posterior intermediate mesoderm and early coelomic epithelium (CE) are indicated by yellow and blue dotted lines, respectively. Left, medial; right, lateral. Bar, 100 μm.

(**C**) IF images of the neighborhood section of (**B**) for WT1 (green) merged with DAPI (white) (top) or FOXF1 (red) (bottom). $FOXF1^+$ LPM/SpM (lateral plate mesoderm/splanchnic mesoderm) is outlined by a red dotted line. Co, coelom. Bar, 100 μm.

(**D**) IF images of the sections taken at the indicated levels, stained with WT1 (green), GATA4 (cyan) and DAPI (white). The white dotted lines outline paraxial mesoderm/somites (PM/Sm). Ms, mesonephros; Me, mesentery; WD, Wolffian duct. Bar, 100 μm.

(**E**) ISH images of sections taken at the indicated levels shown in (**A**) for *GATA4* (red) merged with DAPI (white).

(**F**) (left) Schematic of a 4 wpf human embryo and a transverse section. Approximate axial levels where transverse sections were taken for ISH are indicated by red lines. (right) ISH images of embryos at the indicated stages for *GATA4* (red) merged with DAPI (white). The region highlighted by the black dotted line in the left schematic are shown. $NR5A1^+$ or $NR5A1^-$ CE confirmed by IF on adjacent sections are outlined by yellow or white dotted lines, respectively. Of note, $GATA4^+$ regions are present within CE overlying the ventral aspect of the mesonephros and proximal mesentery. Bar, 100 μm.

Supplementary Figure 2, Cheng et al.

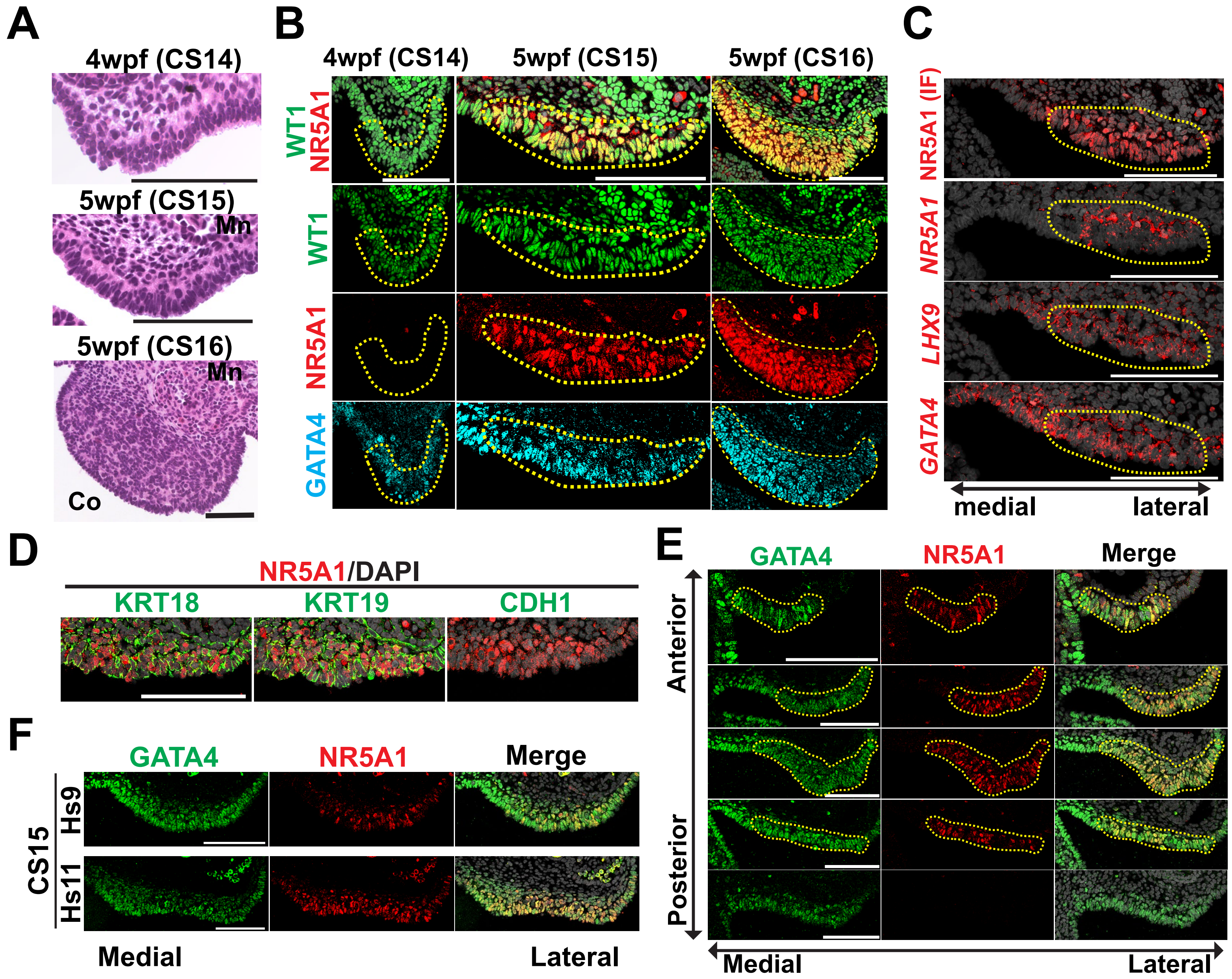

Fig. S2. Specification of the gonads in humans.

(**A**) Representative H&E images of developing human gonads at the indicated stages. Of note, pseudostratification of CE is already evident at CS14. Left, medial; right, lateral. Bar, 100 μm.

(**B**) IF images of emerging gonads (outlined by yellow dotted lines) from posterior sections at the indicated stages for WT1 (green), NR5A1 (red) and GATA4 (cyan). Merged images of WT1 and NR5A1 with DAPI (white) are shown at the top. Left, medial; right, lateral. Bar, 100 μm.

(**C**) ISH images of serial sections of the coelomic angle from a 5 wpf embryo (CS15) for the indicated markers (red) merged with DAPI (white). IF for NR5A1 on the neighboring section is also shown at the top. The NR5A1$^+$ gonad progenitor is outlined by yellow dotted lines. Bar, 100 μm.

(**D**) IF images of the gonad progenitor from the same embryo as in (**C**) for the indicated markers (green) merged with NR5A1 (red) and DAPI (white). Bar, 100 μm.

(**E**) Transverse sections of the gonad progenitor from an embryo at 5 wpf (CS15) from different axial levels along the anterior-posterior axis, stained with GATA4 (green) and NR5A1 (red), and merged with DAPI (white). Of note, the NR5A1$^+$ regions outlined by yellow lines are encompassed by GATA4$^+$ regions. Bar, 100 μm.

(**F**) IF images of the gonad progenitors from two embryos at CS15 (Hs9 and Hs11) stained with GATA4 (green) and NR5A1 (red), and merged with DAPI (white). Bar, 100 μm.

Supplementary Figure 3, Cheng et al.

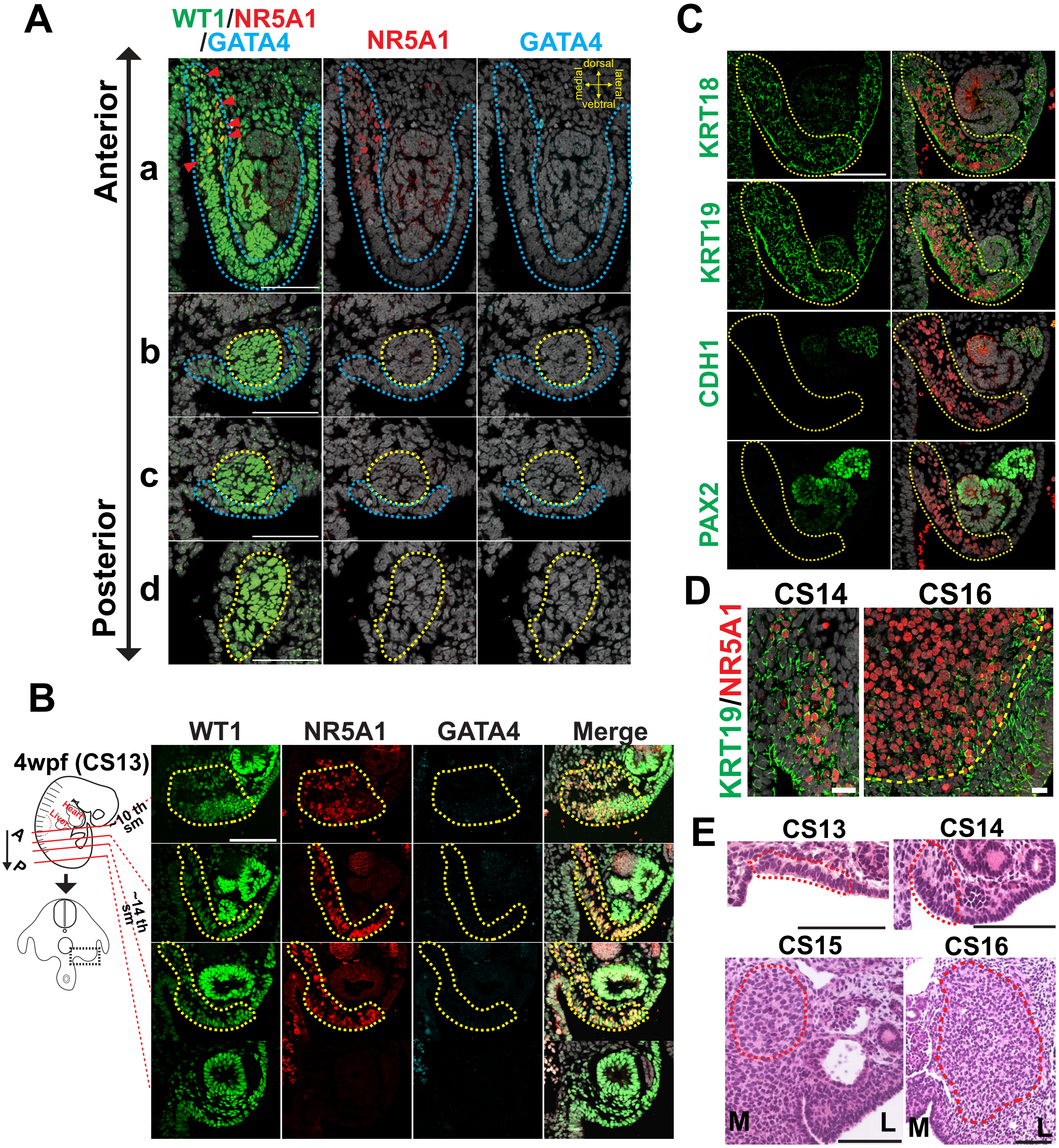

Fig. S3. Specification of the adrenal cortex in humans.

(**A**) IF images of the sections of the embryo at 3wpf (CS12, same as one used in fig. S1A), stained with WT1 (green), GATA4 (cyan), NR5A1 (red) and DAPI (white). Sections were taken from the embryo at the levels shown in fig. S1A (neighborhood sections of those used in fig. S1D). Arrowheads indicate $GATA4^{-}NR5A1^{+}$ emerging adrenogenic coelomic epithelium (CE).

(**B**) (left) Schematic of a human embryo at 4 wpf (CS13), indicating planes where the transverse sections were taken. (right) IF images of these sections for WT1 (green), NR5A1 (red) and GATA4 (cyan), merged with DAPI (white). The yellow dotted lines outline the $NR5A1^{+}$ adrenogenic CE. Of note, $NR5A1^{+}$ adrenogenic CE is seen only at the anterior portion of the embryo (axial levels between approximately the 10th somites just beneath the posterior edge of the liver/anterior limb bud and the 14th somites). Bar, 100 μm.

(**C**) IF images of the coelomic angle (embryos: CS13) for the indicated markers (green) (left). Merged images with NR5A1 (red) and DAPI (white) are shown (right). Bar, 100 μm.

(**D**) IF images of the adrenocortical cells at the migration (CS14, left) or organization stage (CS16, yellow dotted line, right) for KRT19 (green) and NR5A1 (red), merged with DAPI (white). Bar, 20 μm.

(**E**) H&E-stained sections showing the adrenogenic CE of predominantly pre-migratory (top left) and migratory stages (top right), and the adrenal primordium (CS15, bottom left; CS16, bottom right), which are outlined by red dotted lines. M, medial; L, lateral. Bar, 100 μm.

Supplementary Figure 4, Cheng et al.

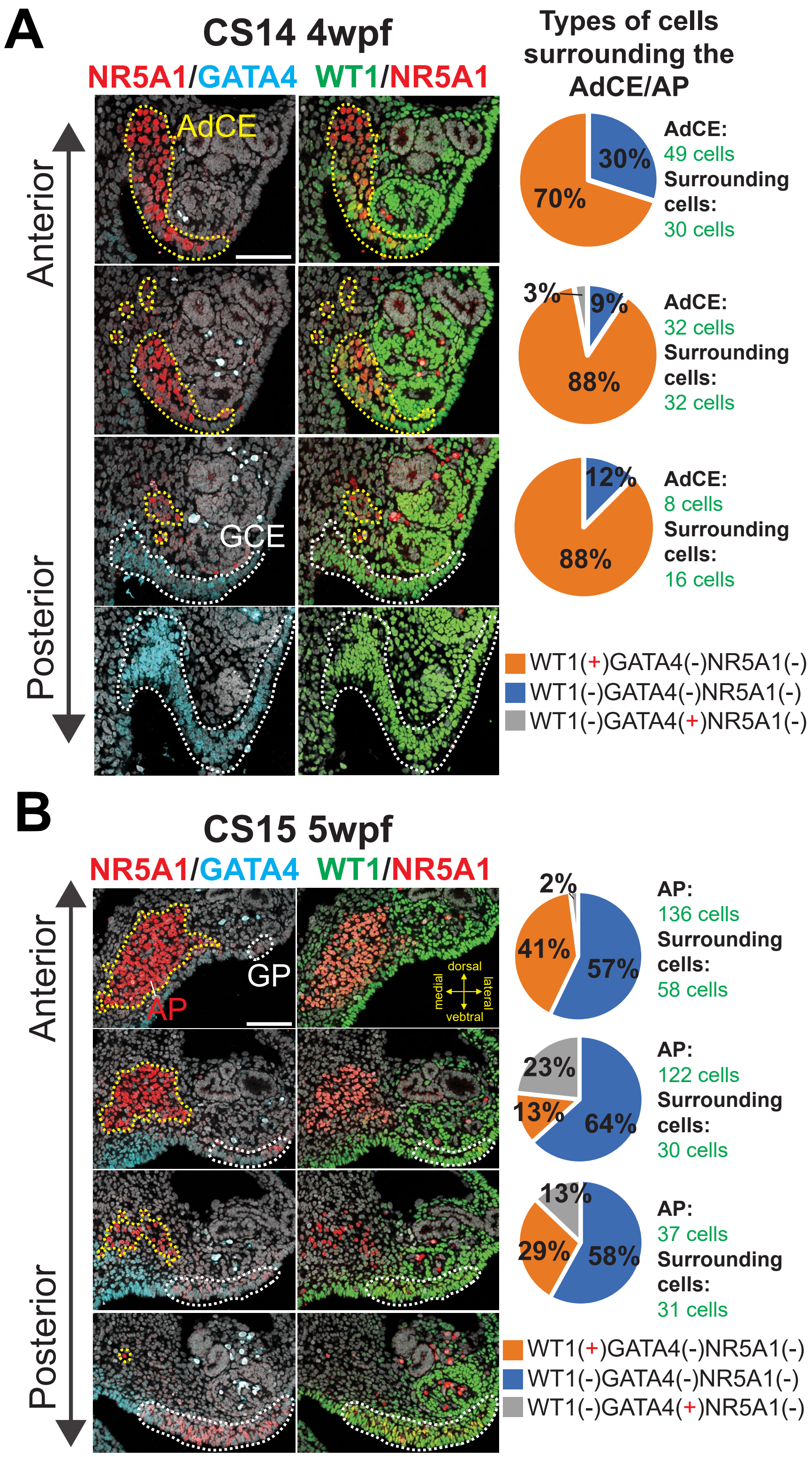

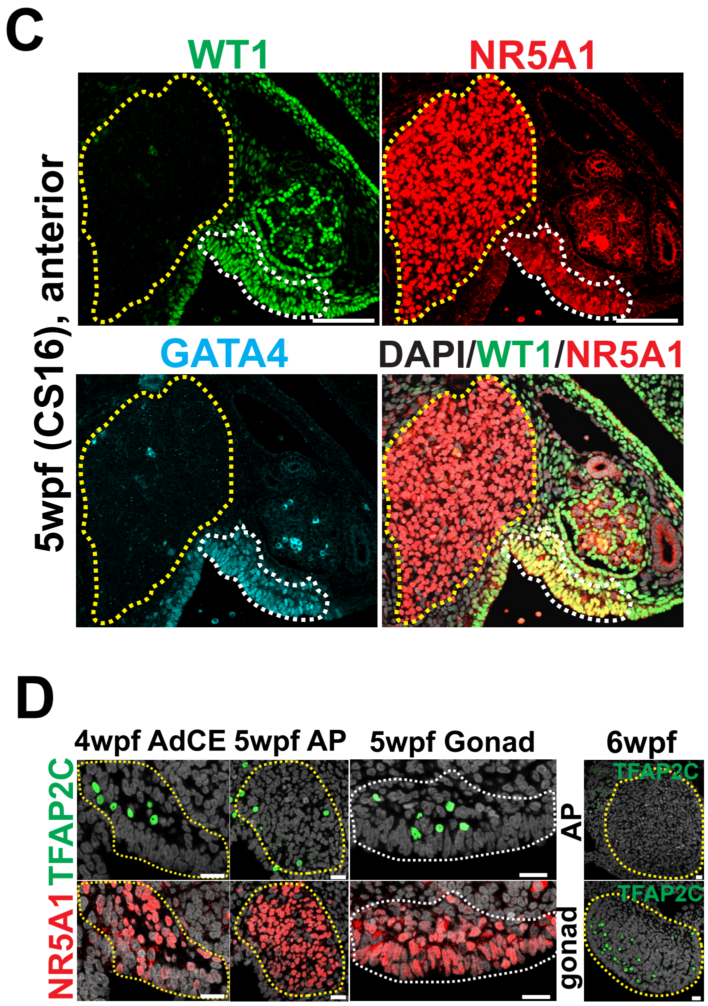

E

	4wpf	5wpf	6wpf
AdCE/AP	8.7% (13/149)	20% (32/163)	0% (0/246)
Gonad	6% (9/149)	42% (68/163)	52% (128/246)
Mesentery	38% (56/149)	28% (46/163)	13% (33/246)
Gut	46% (68/149)	3% (5/163)	2% (5/246)
Peri-aorta/ Mesonephros	2% (3/149)	12% (7/163)	33% (80/246)

Fig. S4. Spatial orientation of adrenocortical and gonadal lineages in humans.
(**A, B**) (left) IF images of the coelomic angle taken from different positions along anterior-to-posterior axis from a 4 wpf (CS14) (**A**) or 5 wpf human embryo (CS15) (**B**) for WT1 (green), NR5A1 (red), GATA4 (cyan) and DAPI (white). Merged images of indicated proteins are shown. Left, medial; right, lateral. Bar, 75 μm. GCE, gonadogenic coelomic epithelium; GP, gonad progenitors; AdCE, adrenogenic coelomic epithelium; AP, adrenal primordium. (right) Pie charts showing % of types of cells surrounding the AdCE/AP. The total number of indicated cell types are also shown on the right. The yellow and white dotted lines outline the AdCE/AP and GCE/GP, respectively.
(**C**) IF images of the anterior section taken from a 5 wpf human embryo (CS16), stained with WT1 (green), NR5A1 (red), GATA4 (cyan). A merged image of WT1, NR5A1 and DAPI (white) is also shown. Bar, 100μm.
(**D**) IF images of human embryos (4–6 wpf, transverse sections) at the indicated regions for TFAP2C (primordial germ cells [PGC] marker, green) and NR5A1 (red), merged with DAPI (white).
(**E**) Distribution of PGCs in different anatomic locations of human embryos at 4–6 wpf, assessed in transverse sections stained with TFAP2C (PGC marker), NR5A1 (markers for the AdCE, AP, and gonads) and GATA4 (gonad marker), merged with DAPI. The percentage (number) of PGCs in each anatomic location for all counted PGCs is shown.

Supplementary Figure 5, Cheng et al.

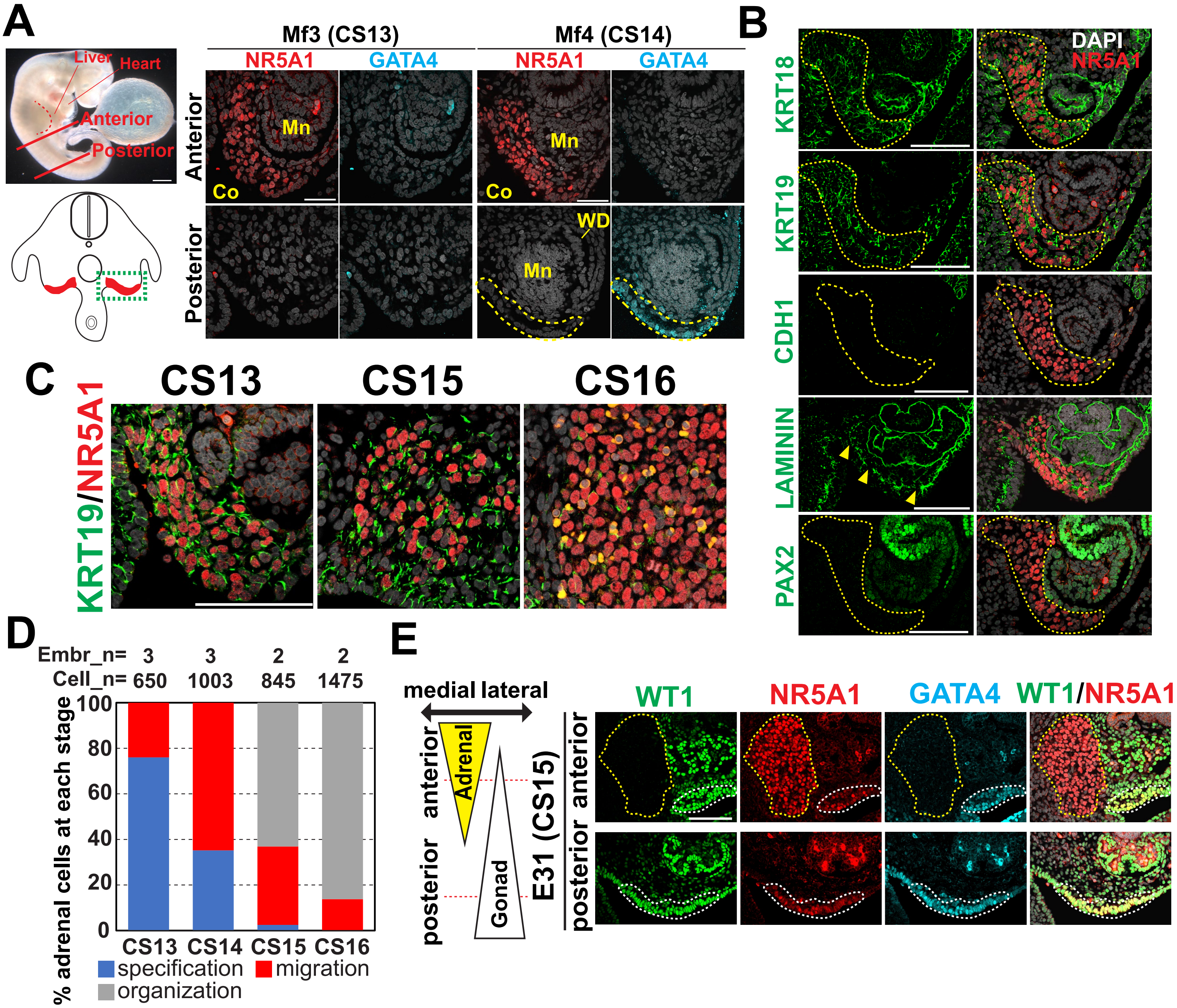

Fig. S5. Specification of the adrenal cortex in cynomolgus monkeys.
(**A**) (left top) Brightfield image of a cynomolgus embryo at CS13. The red dotted line denotes the forelimb bud. The red solid lines indicate the approximate planes where the transverse sections were obtained for IF analysis. Bar, 1 mm. (left bottom) Schematic illustration of transverse sections. Dotted lines outline the coelomic angle where IF images were taken. (right) IF images of the coelomic angle from cynomolgus embryos at CS13 (left, Mf3) and CS14 (right, Mf4) for NR5A1 (red) and GATA4 (cyan), merged with DAPI (white). Co, coelom; Mn, mesonephros; WD, Wolffian duct. Bar, 50 μm.
(**B**) IF images of the coelomic angle (embryos: CS13) for the indicated markers (green) (left). Merged images with NR5A1 (red) and DAPI (white) are shown (right). The yellow dotted lines outline NR5A1^{+} adrenogenic CE. The arrowheads denote regions with basement membrane disruption. Bar, 100 μm.
(**C**) IF images of the adrenocortical cells predominantly in the specification (CS13, left), migration (CS15, middle) or organization stage (CS16, right) for KRT19 (green) and NR5A1 (red), merged with DAPI (white). Bar, 50 μm.
(**D**) Percentage of adrenocortical cells at the indicated morphogenetic stages (specification, migration and organization) for each embryonic stage. Numbers of embryos (Embr_n) and counted cells (cell_n) are shown.
(**E**) (left) Schematic depicting the topological orientation of the adrenal and gonadal linage in a cynomolgus embryo at E31 (CS15). The red dotted lines denote the approximate regions where the sections were made. (right) IF images of the coelomic angles at the representative regions for WT1 (green), NR5A1 (red) and GATA4 (cyan), and merged images for WT1, NR5A1 and DAPI. The yellow and white dotted lines outline the adrenal primordium and gonad progenitor, respectively. Bar, 100 μm.

Supplementary Figure 6, Cheng et al.

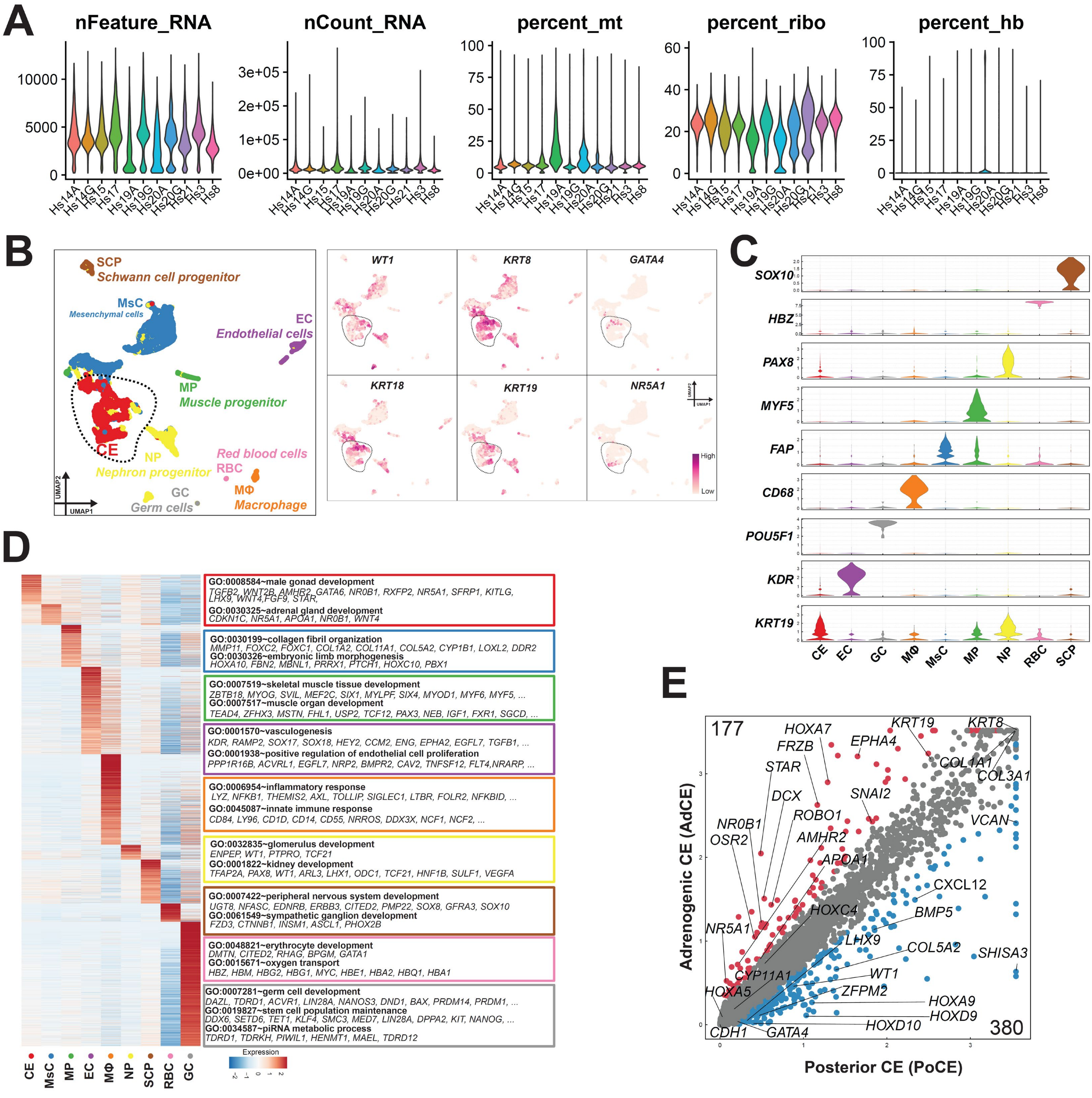

Fig. S6. Quality control of scRNA-seq data and analyses of cell types in the human urogenital ridge at 4 wpf.
(**A**) Quality control analyses of all human samples used in this study.
(**B**) (left) UMAP plot showing different cell clusters in a human embryo at 4 wpf. Cell clusters are annotated on the basis of marker genes. A cluster representing the CE is encircled and isolated for sub-clustering in Fig. 3. (right) Expression of key marker genes for CE. EC, endothelial cells; GC, germ cells; MΦ, macrophage; MsC, mesenchymal cells; MP, muscle progenitor; NP, nephron progenitor; RBC, red blood cells; SCP, Schwann cell progenitor.
(**C**) Violin plot showing the expression of key marker genes in the indicated cell types used for annotating cell clusters in (**B**).
(**D**) (left) Heatmap showing differentially expressed genes (DEGs) among cell clusters. (right) Enriched GO terms and representative genes. Gene expression is Z-score normalized in each row.
(**E**) Scatterplot of pair-wise comparison between adrenogenic CE and posterior CE. DEGs defined as genes with log2 fold change >1, *p*-value <0.01 and FDR <0.01 are shown in colored dots. Key genes and the numbers of DEGs are shown.

Supplementary Figure 7, Cheng et al.

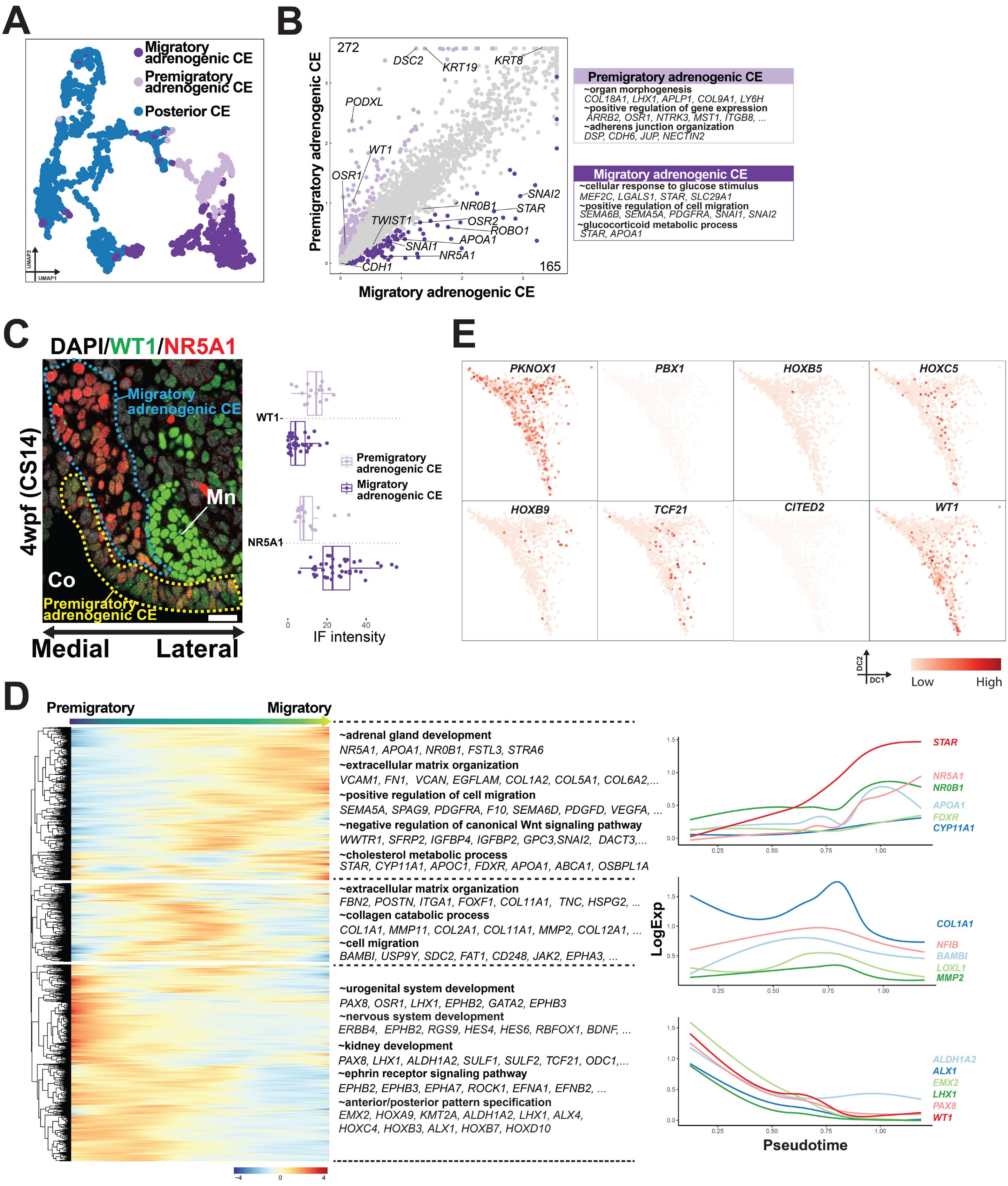

Fig. S7. Gene expression dynamics during the migration of adrenogenic coelomic epithelium.
(**A**) Subclustering of the coelomic epithelium (CE) (defined in Fig. S6B) into posterior CE, premigratory adrenogenic CE and migratory adrenogenic CE, projected on a UMAP plot.
(**B**) (left) Scatterplot of pair-wise comparison between premigratory adrenogenic CE and migratory adrenogenic CE. DEGs defined as genes with log2 fold change >1, *p*-value <0.01 and FDR <0.01 are shown in colored dots. Key genes and the numbers of DEGs are shown. (right) List of DEGs and enriched GO terms.
(**C**) (left) Merged IF image for WT1 (green), NR5A1(red) and DAPI (white), showing the histologic distribution of premigratory adrenogenic CE and migratory adrenogenic CE in a transverse section of a 4 wpf human embryo. (right) Quantification of fluorescence intensity, showing that the adrenogenic CE loses WT1 while gaining NR5A1 expression during the transition.
(**D**) (left) Transcriptome dynamics from premigratory adrenogenic CE to migratory adrenogenic CE, corresponding to Fig. 3D. The top 2000 high variable genes are hierarchically clustered with different patterns along pseudotime. Enriched GO terms are listed at right. (right) Expression dynamics of key variable genes in each cluster at left, aligned along pseudotime.
(**E**) Expression of key genes involved in early adrenocortical development in mice projected on the plot used in Fig. 3D.

Supplementary Figure 8, Cheng et al.

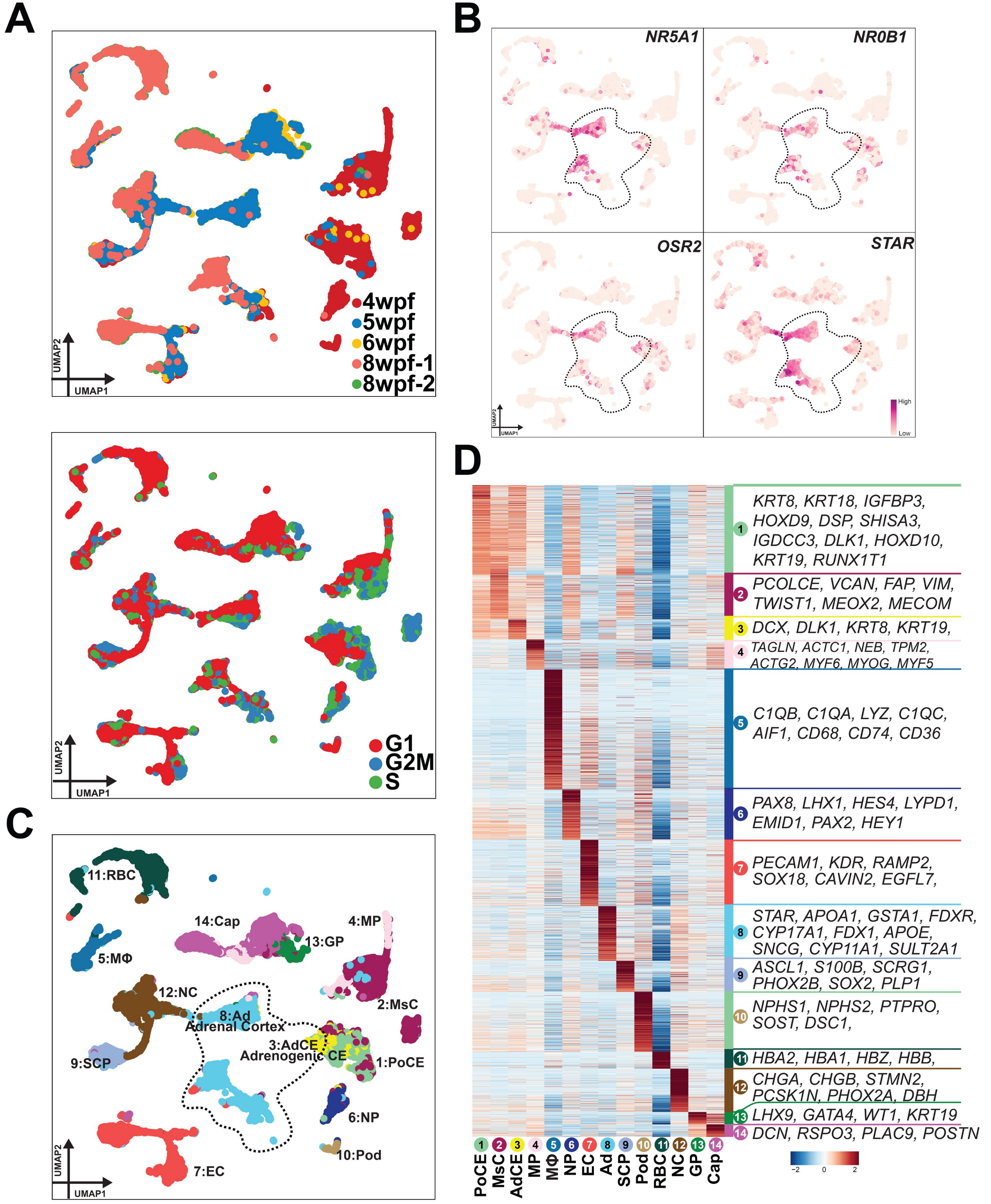

Fig. S8. scRNA-seq analyses to define adrenal lineages in human embryos.
(**A**) UMAP plot showing all cells from human embryonic adrenal glands (5–8 wpf) and a urogenital ridge (4 wpf) used to isolate the adrenal lineage for the trajectory analysis in Fig. 3F. Each dot represents a single cell, colored according to sample origin (top) and cell cycle scoring (bottom).
(**B**) Expression of key marker genes projected on a UMAP plot to define adrenal lineages. Cells outlined by dotted lines are annotated as having adrenal lineage in (**C**) and were isolated for further sub-clustering in Fig. 3F.
(**C**) Cell clusters and their annotations, projected on the UMAP plot used in (**A, B**). PoCE, posterior CE; MsC, mesenchymal cells; AdCE, adrenogenic CE; MP, muscle progenitor; MΦ, macrophage; NP, nephron progenitor; EC, endothelial cells; Ad, adrenal cortex; SCP, Schwann cell progenitor; Pod, podocytes; RBC, red blood cells; NC, neuroendocrine cells; GP, gonad progenitor; Cap, capsular cells.
(**D**) Heatmap of DEGs among the cell clusters. Representative genes are listed at right. Gene expression is Z-score normalized in each row.

Supplementary Figure 9, Cheng et al.

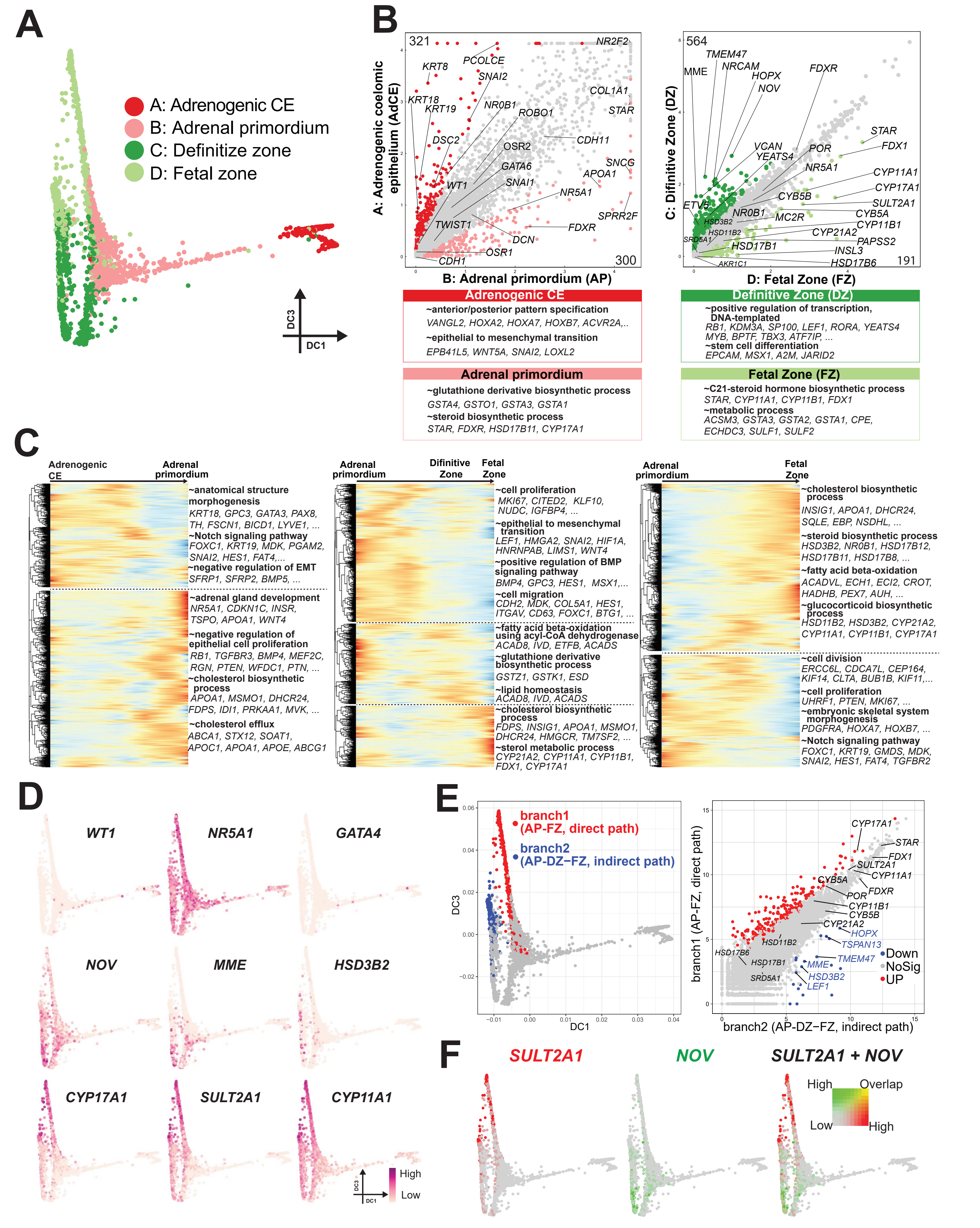

Fig. S9. Gene expression dynamics during early adrenal development in humans.
(**A**) 2D diffusion map using the first and third diffusion components and the same clusters as in Fig. 3F.
(**B**) (top) Scatterplots comparing the averaged gene expression levels between adrenogenic CE (cluster A) and adrenal primordium (cluster B) (left), or definitive zone (cluster C) and fetal zone (cluster D) (right). Differentially expressed genes (DEGs) upregulated more than 2-fold (p-value <0.01 and FDR <0.01) are shown. Key genes are annotated. (bottom) GO analyses of DEGs. Representative genes in each GO category are shown.
(**C**) Heatmap showing transcriptomic dynamics during early adrenal development. Each row represents a gene, and each column represents a cell, which is ordered by the pseudotime defined in Fig. 3H. Trajectories partitioned in three branches are shown separately (adrenogenic CE to adrenal primordium, left; adrenal primordium to fetal zone via definitive zone, middle; adrenal primordium to fetal zone, right). The top 2000 variable genes are hierarchically clustered. Representative genes and their enriched GO terms for variable genes clustered on the basis of expression patterns are shown at right.
(**D**) Expression of key genes projected on the 2D diffusion map, as in (**A**).
(**E**) (left) 2D diffusion map showing fetal zone cells on direct (branch 1, red) and indirect paths (branch 2, blue), as in (**A**). The fetal zone cells (cluster D in [**A**]) were assigned to either branch 1 (855 cells) or 2 (390 cells) by fitting principal curves to the data using ElPiGraph.R. (right) Scatterplots comparing the averaged gene expression levels between the fetal zone cells on blanch 1 (x-axis) and blanch 2 (y-axis). DEGs upregulated more than 2-fold (p-value <0.01 and FDR <0.01) are shown. Key markers for the fetal zone and definitive zone are shown.
(**F**) Expression of *SULT2A1* (fetal zone marker) and *NOV1* (definitive zone marker) projected on the 2D diffusion map, as in (**A**).

Supplementary Figure 10, Cheng et al.

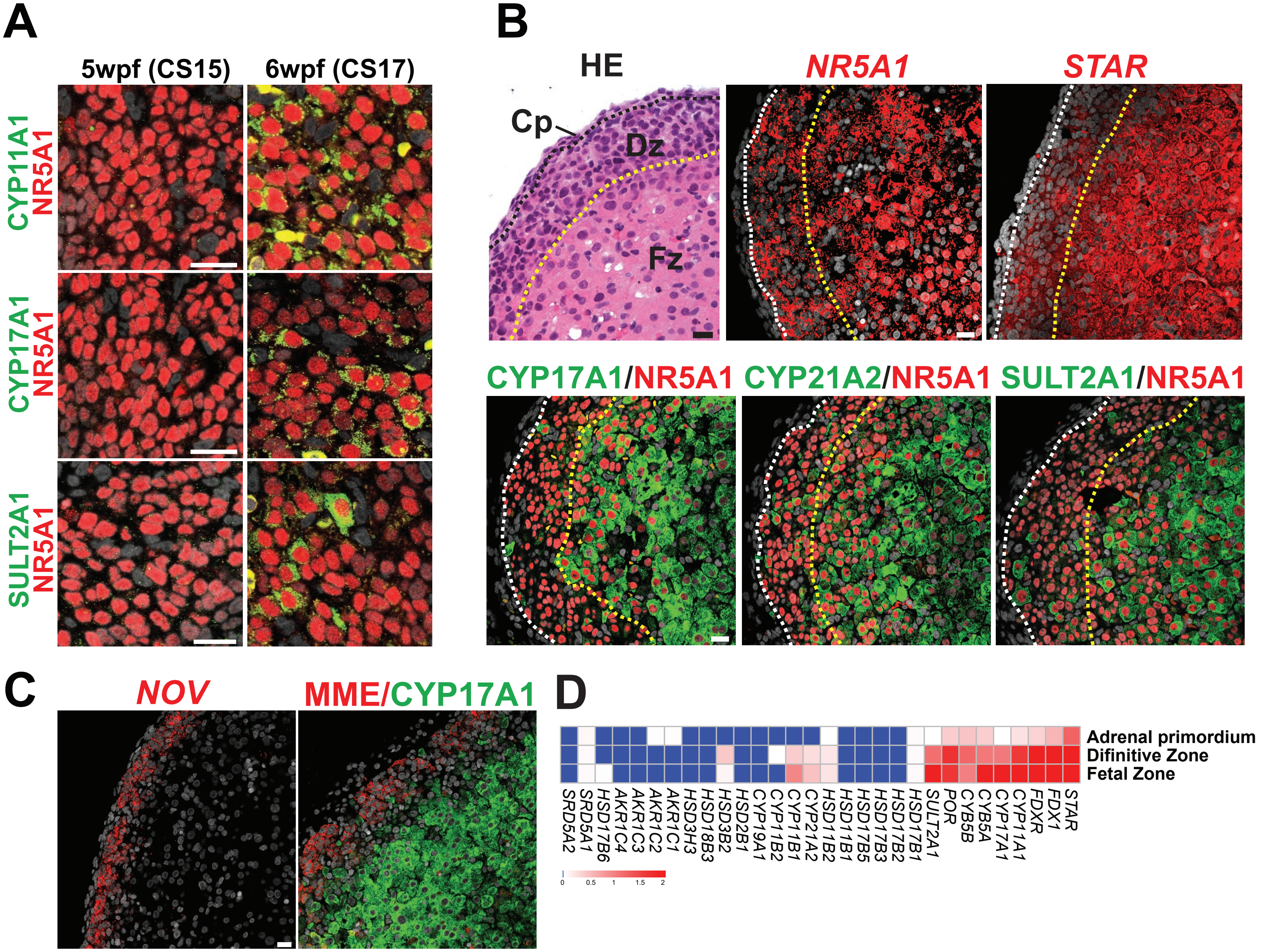

Fig. S10. Initiation of steroidogenesis and zonation in developing human adrenal glands.
(**A**) IF of transverse sections of human embryos at the indicated stage for NR5A1 (red) and key steroidogenesis genes (CYP11A1, CYP17A1 and SULT2A) (green) merged with DAPI (white). Bar, 20 μm.
(**B**) (top left) H&E image of a cross section of human fetal adrenals at 8 wpf. Cp, capsule; Dz, definitive zone; Fz, fetal zone. (top right) ISH images of the same embryo for *NR5A1* or *STAR* (red), merged with DAPI (white). (bottom) IF images of the same embryo for key steroidogenesis genes (CYP21A2, CYP17A1 and SULT2A) (green) merged with NR5A1 (red) and DAPI (white). The white dotted lines separate the capsule and definitive zone. The yellow dotted lines separate the definitive zone from the fetal zone. Bar, 20 μm.
(**C**) (left) ISH on a section of the adrenals at 8 wpf, showing specific expression of *NOV* (Red) in the definitive zone. (right) IF on the neighboring section for MME (red) and CYP17A1 (green), highlighting the definitive zone and fetal zone, respectively. Merged images with DAPI are shown. Bar, 20 μm.
(**D**) Heatmap showing the averaged expression of key genes associated with steroidogenesis in the adrenal primordium, definitive zone and fetal zone. Color indicates log normalized expression.

Supplementary Figure 11, Cheng et al.

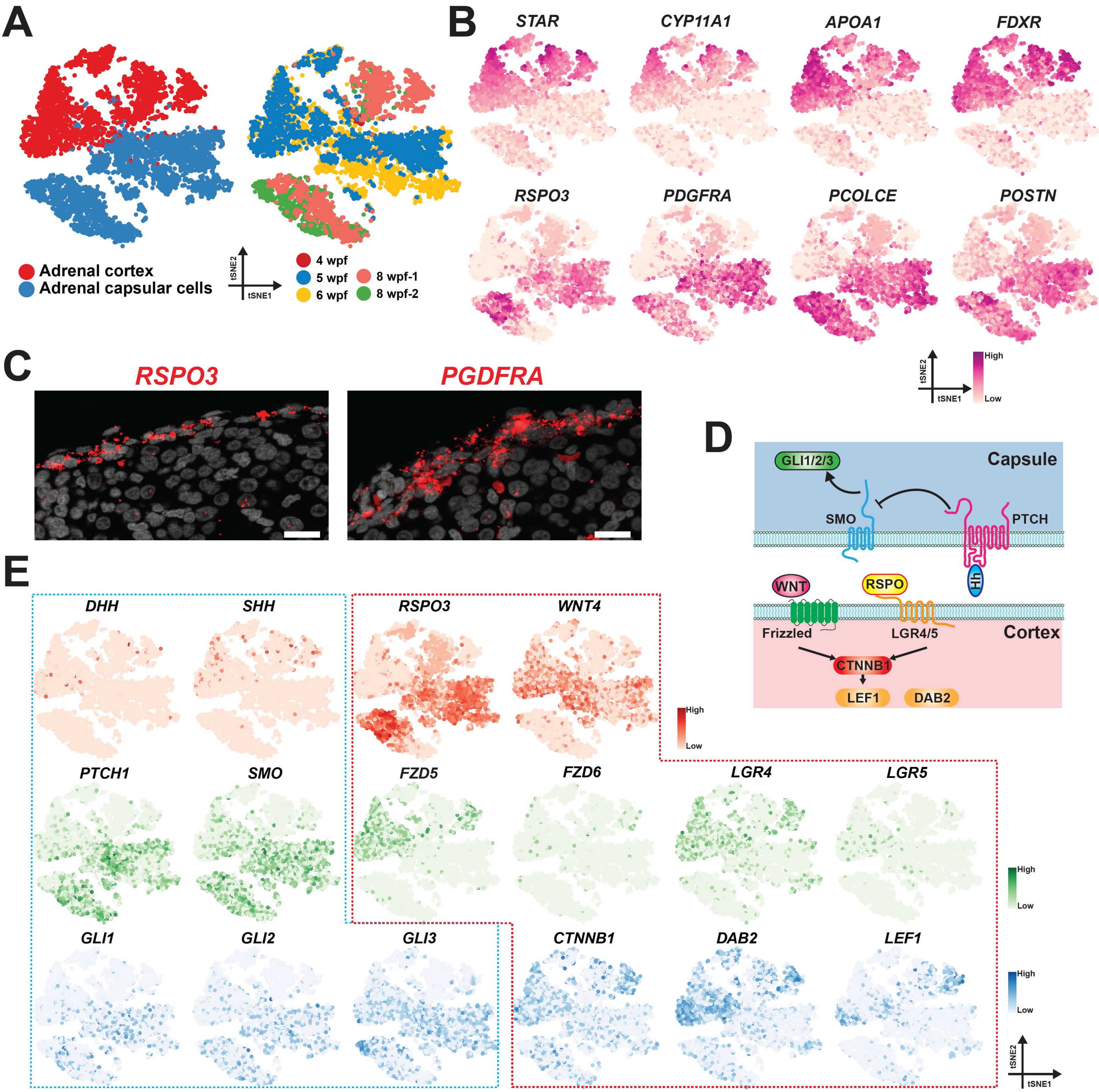

Fig. S11. Receptor-ligand interaction between the cortex and capsule in human fetal adrenals.
(**A**) (left) Adrenal cortex (clusters 8 in Fig. S8C) and capsular cells (cluster 14 in Fig. S8C), projected on the t-distributed stochastic neighbor embedding (tSNE) plot. (right) Sample origins projected on the same tSNE plot.
(**B**) Expression of key marker genes used for annotation of the adrenal cortex and capsular cells.
(**C**) ISH on the paraffin section of human fetal adrenal cortex (8 wpf) for *RSPO3* and *PDGFRA* (red), merged with DAPI (white). Bar, 20 μm.
(**D**) Diagram depicting the interactions between the cortex and the capsule.
(**E**) Expression of ligands (top), receptors (middle) and target genes (bottom), projected on the tSNE in (A). Hedgehog and WNT pathways are outlined in blue and red dotted lines, respectively.

Supplementary Figure 12, Cheng et al.

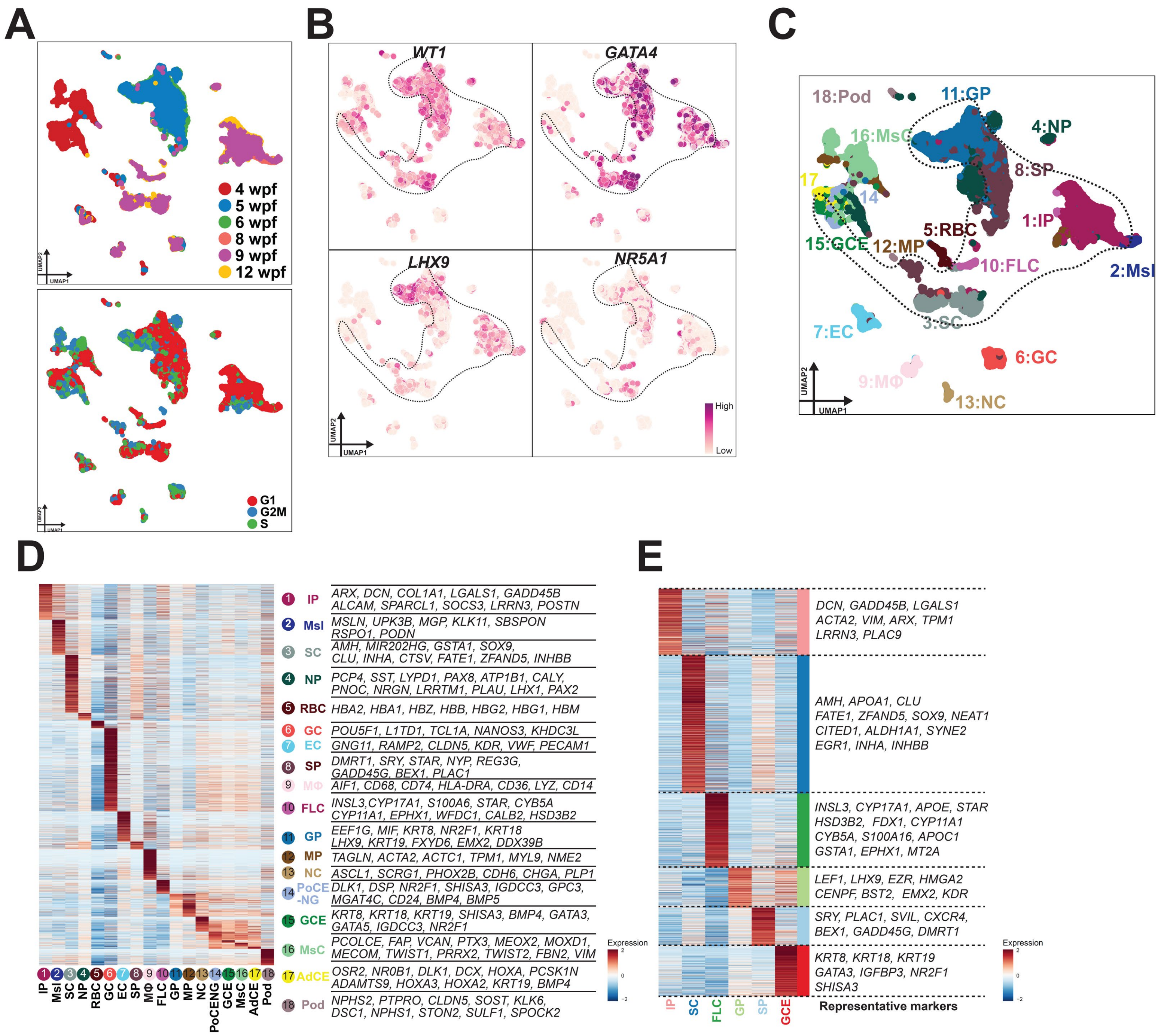

Fig. S12. scRNA-seq analyses to define gonadal lineages in human embryos.
(**A**) UMAP plot showing all cells from human embryonic testicular cells (5–12 wpf) and the urogenital ridge (4 wpf) used to isolate the testicular lineage for the trajectory analysis in Fig. 4A. Each dot represents a single cell, colored according to its sample origin (top) and cell cycle scoring (bottom).
(**B**) Expression of key marker genes of the gonadal (testicular) lineage, projected on the UMAP plot in (**A**). Cells in circles are annotated as having gonadal lineage and isolated for trajectory analysis in Fig. 4A.
(**C**) Cell clusters and their annotations, projected on a UMAP plot. Cells are colored according to cell cluster. IP, interstitial progenitors; Msl, mesothelium; SC, Sertoli cells; NP, nephron progenitors; RBC, red blood cells; GC, germ cells; EC, endothelial cells; SP, Sertoli cell progenitors; MΦ, macrophage; FLC, fetal Leydig cells; GP, gonad progenitors; MP, muscle progenitors; NC, neuroendocrine cells; GCE, gonadogenic CE; MsC, mesenchymal cells; Pod, podocytes.
(**D**) Heatmap showing the averaged expression pattern of DEGs among the cell clusters (left) and key genes (right). Gene expression is Z-score normalized in each row. PoCE-NG, non-gonadal posterior CE; AdCE, adrenogenic CE.
(**E**) Heatmap showing the averaged expression pattern of DEGs among different cell clusters of gonadal lineages identified in Fig. 4A (left) and representative genes (right). Gene expression is Z-score normalized by row.

Supplementary Figure 13, Cheng et al.

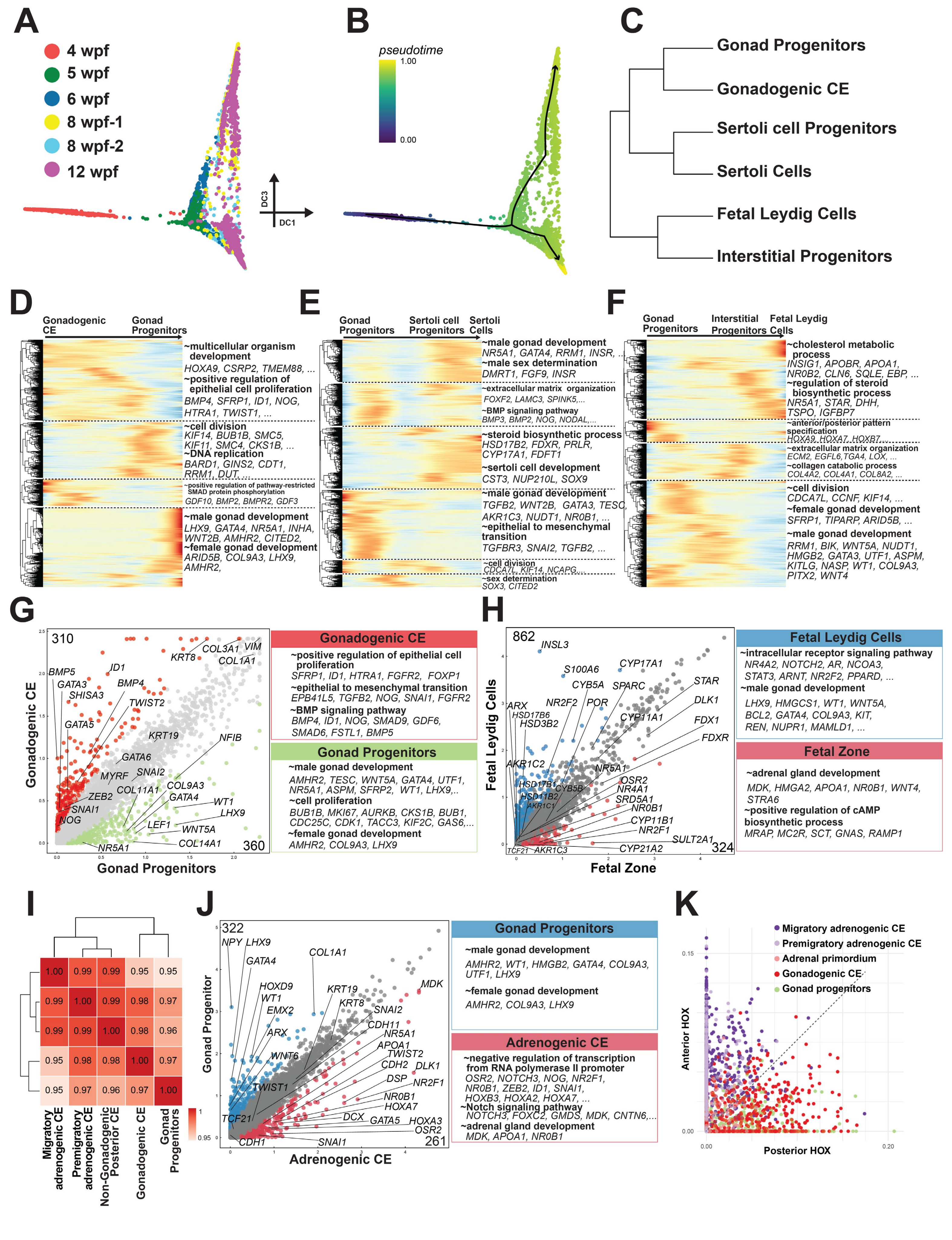

Fig. S13. Characterization of developing gonadal lineages and comparison with adrenal lineages in humans.

(**A**) Projection of sample origins on the diffusion map used in Fig. 4A.

(**B**) Projection of pseudotime and fitted principal curves on the diffusion map.

(**C**) Hierarchical clustering dendrogram showing the relationships among the cell clusters. The correlation matrix was computed with the Spearman method.

(**D, E, F**) Heatmap showing gene expression dynamics along lineage trajectories (defined in **B**) from gonadogenic CE to gonad progenitors (**D**), from gonad progenitor to Sertoli cells (**E**), and from gonad progenitors to fetal Leydig cells (**F**). Each row is a gene, each column is a cell, and cells are ordered by pseudotime as in (**B**). The top 2000 high variable genes are hierarchically clustered. Gene expression is Z-score normalized by row.

(**G**) Scatterplot comparing the averaged gene expression levels between gonadogenic CE and gonad progenitors. DEGs upregulated in gonadogenic CE (red) and gonad progenitors (green) (2-fold change, *p*-value <0.01 and FDR <0.01) are highlighted, and key genes are annotated. (right) GO analyses of the DEGs. Representative genes in each GO category are shown.

(**H**) (left) Scatterplot comparison of the averaged values of DEGs between fetal Leydig cells and the fetal zone. Genes upregulated by more than 2-fold (*p*-value <0.01, FDR <0.01) in the indicated cell clusters are plotted with colors. Key genes and the numbers of DEGs are shown. (right) Over-represented GO terms and representative genes in each GO category are shown.

(**I**) Spearman correlation and hierarchical clustering of the cell clusters, showing that the gonad progenitor is close to gonadogenic CE rather than adrenogenic CE.

(**J**) (left) Scatterplot comparison of the averaged values of DEGs between gonad progenitors and adrenogenic CE. Genes upregulated by more than 2-fold (*p*-value <0.01, FDR <0.01) in the indicated cell clusters are plotted with colors. Key genes and the numbers of DEGs are shown. (right) Over-represented GO terms and representative genes in each GO category are shown.

(**K**) Area under the curve (AUC) scoring of *HOX* genes in the indicated cell types. Each dot represents a single cell, colored according to the cell clusters annotated in Fig. 3D, F and 4A. Cells in the gonadal lineage (gonadogenic CE, gonad progenitors) are biased toward higher posterior HOX scores, whereas cells in the adrenal lineage (migratory adrenogenic CE, premigratory adrenogenic CE, adrenal primordium) are biased toward higher anterior HOX scores.
